# Conical and sabertoothed cats as an exception to craniofacial evolutionary allometry

**DOI:** 10.1038/s41598-023-40677-6

**Published:** 2023-08-21

**Authors:** Davide Tamagnini, Margot Michaud, Carlo Meloro, Pasquale Raia, Leopoldo Soibelzon, P. Sebastián Tambusso, Luciano Varela, Luigi Maiorano

**Affiliations:** 1 Department of Biology and Biotechnologies “Charles Darwin”, University of Rome “La Sapienza”, Zoology Building, Viale dell’Università 32, 00185 Rome, Italy; 2 Museum of Zoology, Sapienza Museum Centre, University of Rome “La Sapienza”, Rome, Italy; 3 Evolution & Diversity Dynamics Lab, University of Liège, Liege, Belgium; 4 School of Biological and Environmental Sciences, Research Centre in Evolutionary Anthropology and Palaeoecology, Liverpool John Moores University, Liverpool, UK; 5 Dipartimento di Scienze della Terra, dell’Ambiente e delle Risorse, University of Naples Federico II, Naples, Italy; 6 División Paleontología Vertebrados, Museo de La Plata, Facultad de Ciencias Naturales y Museo, Universidad Nacional de La Plata, Paseo del Bosque s/n, 1900 La Plata, Argentina; 7 Consejo Nacional de Investigaciones Científicas y Tecnicas (CONICET), Godoy Cruz 2290, C1425FQB CABA, Argentina; 8 Departamento de Paleontología, Facultad de Ciencias, Universidad de la República, Iguá 4225, 11400 Montevideo, Uruguay; 9 Departamento de Canelones, Servicio Académico Universitario y Centro de Estudios Paleontológicos (SAUCE-P), Universidad de la República, Santa Isabel s/n, 91500 Sauce, Uruguay

**Keywords:** Evolution, Palaeontology, Biomechanics

## Abstract

Among evolutionary trends shaping phenotypic diversity over macroevolutionary scales, CREA (CRaniofacial Evolutionary Allometry) describes a tendency, among closely related species, for the smaller-sized of the group to have proportionally shorter rostra and larger braincases. Here, we used a phylogenetically broad cranial dataset, 3D geometric morphometrics, and phylogenetic comparative methods to assess the validity and strength of CREA in extinct and living felids. To test for the influence of biomechanical constraints, we quantified the impact of relative canine height on cranial shape evolution. Our results provided support to CREA at the family level. Yet, whereas felines support the rule, big cats, like Pantherinae and Machairodontinae, conform weakly if not at all with CREA predictions. Our findings suggest that Machairodontinae constitute one of the first well-supported exceptions to this biological rule currently known, probably in response to the biomechanical demands and developmental changes linked with their peculiar rostral adaptations. Our results suggest that the acquisition of extreme features concerning biomechanics, evo-devo constraints, and/or ecology is likely to be associated with peculiar patterns of morphological evolution, determining potential exceptions to common biological rules, for instance, by inducing variations in common patterns of evolutionary integration due to heterochronic changes under ratchet-like evolution.

## Introduction

A central goal in present-day research in macroevolution is to unravel the processes determining phenotypic evolutionary trajectories over long-time scales. An important tool to achieve this goal has been suggested to lie in the study of the most influential drivers determining presence, absence, and variations in peculiar patterns of trait evolution, the evolutionary trends^1–3^. Evolutionary trends are defined as persistent and directional changes in the state of one or more quantitative traits through evolutionary time. As such, they characterise evolution at high taxonomic levels and over geological time scales^4–6^. Among such trends, there are the so-called biological rules, which are the results of directional responses to ecological, climatic or biological gradients and occur in a large number of clades^3,7^. Typical examples of biological rules are Bergmann’s, Allen’s or Cope’s rules, which represent directional variations in species traits (e.g., body size or surface-area-to-volume ratio) over latitudinal, elevational or temporal gradients^8–10^. Identifying such phenotypic patterns over macroevolutionary scales can greatly improve our knowledge on how biodiversity is structured through time, for instance, by allowing the realization of models to make inferences on the predictability of evolution in climate change scenarios^11,12^. In this context, highlighting exceptions to these trends allows researchers to identify the different factors that shape phenotypic diversity as well as their interactions, but also stress out factors hitherto not considered. For example, a recent study conducted by Faurby and Araújo^13^ suggested that anthropogenic pressures weaken the body mass to latitude pattern expected to occur under Bergmann’s rule.

When it comes to research on evolutionary trends, the formulation of a new biological rule, known as CRaniofacial Evolutionary Allometry (CREA)^14,15^, recently became a central topic in the field. CREA alleges that, among closely related species, the smaller-sized of the group would appear paedomorphic, possessing proportionally smaller rostra and larger braincases^14,15^. Morphological evolutionary allometry is a ubiquitous phenotypic phenomenon that can be interpreted as a form of integration (i.e., the tendency of multiple traits to covary throughout a biological structure^16^; in line with the concept of epigenetic interaction sensu Waddington^17,18^) and is often maintained by natural selection acting on the genetic and pleiotropic architecture that underlies this evolutionary pattern^19,20^. Because CREA pattern describes the size-shape relationship between different parts of the cranium (i.e., rostrum versus braincase size and shape), the concept is intrinsically linked to that of phenotypic integration^21,22^.

Different factors can maintain or alter the strength of integration at the macroevolutionary scale. For example, strong parallel selection pressures imposed by high biomechanical constraints often result in a higher morphological integration between structures^23,24^. By contrast, ecological specialization towards peculiar niches may also result in a decrease in phenotypic integration due to functional divergences^25–28^. The main evolutionary stimuli and dynamics involved in CREA are yet to be understood^15^. Potential (and non-mutually exclusive) explanations range from the need to face dietary, biomechanical, and metabolic trade-offs^14^, through the existence of genetic correlations between craniofacial morphology and body mass^29^, to the presence of constraints imposed by evo-devo dynamics (which would limit morphological evolution in many directions of the multivariate trait space and would enhance changes along specific lines of least evolutionary resistance^30,31^. Understanding these dynamics is even more important given the analogy between CREA and other evolutionary trends generated via artificial selection, collectively known as the domestication syndrome^15,32–35^.

Following Radinsky^36^, who first tested for the occurrence of evolutionary allometry in the cranium of several mammalian clades, a small number of studies have since addressed the occurrence and strength of CREA within mammalian lineages. These investigations confirmed the validity of CREA within groups ranging from metatherians (e.g., wallabies and kangaroos belonging to the genus *Macropus*^37^; opossums^38,39^ to placentals (e.g., capuchins and squirrel monkeys, Australian rodents, pangolins, and many other clades belonging to all superorders of placental mammals^15,40–42^. Further confirmation was found in other vertebrate clades, such as birds of prey^43^ and temnospondyl amphibians^44^. By contrast, CREA was supported only in a limited number of nonmammalian synapsid fossils^45^.

Within mammals, species belonging to the order Carnivora (henceforth referred to as carnivorans) represent a classic case study for morphological macroevolution thanks to their substantial ecological flexibility, high taxonomic diversity, and remarkable morphological variability^46,47^. Previous investigations on CREA performed within terrestrial carnivorans applying either traditional or geometric morphometric techniques fully supported the presence of this evolutionary trend in mustelids (i.e., weasels, ferrets, and minks^36^, canids (i.e., wolves, wild dogs, and foxes^15,48^, and herpestids (i.e., mongooses^14,15^). Analogous approaches suggested the pattern may be present in living felids, although big cats were hypothesised to be a partial exception to CREA as a result of potentially divergent pressures in cranial shape evolution acting between their two subclades, the large-sized genus *Panthera* and the medium-sized and sabertooth-like genus *Neofelis*^49,50^.

**Figure 1:**
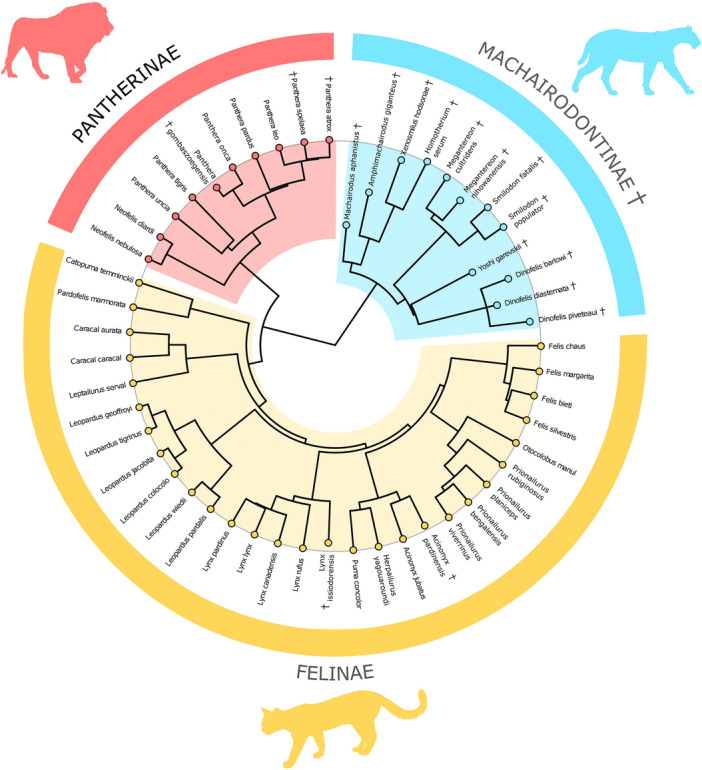
Circular dendrogram representing the phylogeny from Piras et al.^64^ showing the distribution of each taxon in our allometric analyses on living and extinct felids. The subfamily Felinae (i.e., small and medium conical-toothed cats) is represented in yellow, the subfamily Pantherinae (i.e., large conical-toothed cats) in red, and the subfamily Machairodontinae (i.e., sabertoothed cats) in light blue. The symbol † represents an extinct species.

Due to their hypercarnivorous lifestyle^51,52^, the morphological evolution of felids has always caught the interest of evolutionary biologists^53,54^. This also applies to paleontological research, in which sabertoothed cats (i.e., extinct subfamily Machairodontinae) represent one of the most fascinating and renowned groups for ecomorphological studies due to their extremely elongated, laterally-compressed, and curved upper canines that protrude from the mouth when closed^55–58^. Conical-toothed felids (i.e., fossil and living Felinae and Pantherinae—Fig. 1) display higher ecomorphological flexibility as compared to sabertooths, whose fearsome but delicate upper canine have been generally interpreted as an adaptation to a hyperspecialised diet of large ungulates and juvenile proboscideans^59–61^. By considering CREA as a form of integration between the rostral and the braincase modules, felids thus represent an ideal case study for research on this evolutionary trend because of the extreme rostral adaptations occurring in some of their members (i.e., sabertoothed cats) that might have overruled CREA within this group. In particular, CREA analyses within living and fossil felids might also give researchers the opportunity to further clarify if potential exceptions to this evolutionary trend are linked with strong and unusual biomechanical demands occurring in the rostrum (e.g., need to have a wide gape angle in sabertoothed cats in order to attack larger preys).

To date, studies assessing CREA that include paleontological data are extremely rare (but see^36,45^), and none of them relies on modern techniques of 3D morphological quantification (e.g., 3D geometric morphometrics—3D GMM). This knowledge gap persists despite that the inclusion of fossil data has already been suggested to be a key feature for improving the accuracy of allometric analyses, increasing taxonomic sampling, and detecting potential exceptions to CREA^45,50^.

Here, we use 3D GMM on a phylogenetically broad cranial dataset (n = 51 inclusive of fossil and extant species) in combination with phylogenetic comparative methods to assess CREA in the entire family Felidae and its three subfamilies (i.e., Felinae, Pantherinae, and Machairodontinae). To do so, we also explore whether phylogenetic uncertainty may impact whether or to what extent CREA applies. Furthermore, we assess the impact of a proxy for biomechanical performance (i.e., Relative Canine Height—RCH) on cranial shape evolution within felids and its subfamilies. Slater and Van Valkenburgh^62^ already identified RCH as an important factor of craniofacial shape variation in extinct sabertoothed species including not only members of Felidae, but also Nimravidae and Barbourofelidae. Meloro and Slater^63^ confirmed this trait to impact the rostral shape and its level of covariation with the braincase in sabertoothed species. Still, it remains unclear if this pattern applies only to the lateral aspect of crania or also to the ventral and the dorsal regions, and if it can be recognised within monophyletic clades (i.e., Felidae). The combination of these tests aims to answer two meaningful macroevolutionary questions: is CREA a pervasive evolutionary trend throughout the evolutionary history of the monophyletic clade Felidae or sabertoothed cats, with their extreme cranial adaptations, constitute an exception to this biological rule? Does the acquisition of relatively longer upper canines, which determines wider gape angles, have an impact on the cranial shape evolution of felids, potentially invalidating CREA pattern?

## Materials and methods

### Sample and data collection

The morphological sample consisted of 98 felid crania, including almost 90% of the extant species diversity (34 out of 38) and 17 fossil species (12 sabertoothed and five conical-toothed cats). All individuals selected were adults, as assessed by the complete dentition and fusion of their cranial sutures. Sample composition is detailed in SI Appendix, Table S1. For each living species, both sexes were included in the data whenever possible. Using a single (or a few) individual(s) to represent a species is considered adequate in a macroevolutionary analysis involving large interspecific and intergeneric differences^65,66^. When multiple specimens belonging to the same species were available, morphological data were averaged within species, obtaining pooled-sex data (see SI Appendix for sensitivity analyses regarding sexual dimorphism). The vast majority of digital 3D models used in this study was collected by the same operator (DT) employing a digital SLR Nikon D7000 camera attached to a Nikkor 40 mm macro lens to perform photogrammetric reconstructions using the software Agisoft (version 1.6.5). Photogrammetry was applied following guidelines from Falkingham^67^ and Mallison and Wings^68^. Additionally, a small proportion (i.e., c. 25%) of the specimens was either taken from online repositories (i.e., DigiMorph, MorphoSource, Phenome10K, Sketchfab, and digital collections of Primate Research Institute (Kyoto) and Museu de Ciències Naturals (Barcelona)) and/or deriving from materials included in previous studies^69–72^. These 3D models were obtained using photogrammetry, computed tomography or surface laser scanning techniques. Multi-technique datasets including models deriving from the use of all these methods were recently demonstrated to be suitable for assessing the presence and the strength of allometry in macroevolutionary analyses with wide phylogenetic scope^73^. The taxonomy adopted in this study followed the IUCN Red List website (https://www.iucnredlist.org) for living species and previous ecomorphological studies for fossil ones^64^.

### Landmark configurations, digitisation, and geometric morphometrics

The landmark configuration is shown in Fig. S1, and the definition of each landmark is provided in Table S2. Allometric analyses were performed using a set of 30 landmarks (30L configuration—SI Appendix, Tab. S2) at first and then redone including only a subset of 10 landmarks (10L configuration—SI Appendix, Tab. S2, rows with grey background). The former configuration was employed to describe general functional aspects of cranial morphology, whereas the latter was selected to capture more specifically the relative proportions of the rostrum and the braincase.

3D landmark digitisation was performed using the software Stratovan Checkpoint^74^ by a single operator (DT) to avoid inter-operator biases. The repeatability and precision of landmark configurations were tested (SI Appendix). Details about the retrodefomation of distorted fossil specimens and the estimation of missing landmarks are provided in SI Appendix. In order to remove non-shape variation from three-dimensional Cartesian coordinates of landmarks, we employed the Procrustes superimposition method^75,76^. This procedure consists of three steps: the standardization of size, the removal of translational variation, and the minimization of rotational differences^75^. Procrustes superimposition was performed using the software MorphoJ (version 1.06d)^77^. As this study does not focus on the analysis of asymmetries and given the low amount of variance explained by the asymmetric component of shape on the cranial sample (< 5% of the total shape variance), all allometric analyses focused only on the symmetric component of shape variation^78^.

### Allometric and shape versus relative canine height regressions

Cranial evolutionary allometry was tested on a sample of pooled-sex species means by regressing all principal components describing shape variation against the natural logarithm of centroid size, since using the logarithm of size is considered good practice in allometric analyses when the range of size is as large as in our case^22^. All multivariate regressions were performed using the 30L configuration and then repeated using the 10L configuration. The regressions were performed within the family Felidae (51 species) and then replicated within its three subfamilies (i.e., conical-toothed Felinae and Pantherinae—29 and 10 species, respectively; sabertoothed Machairodontinae—12 species).

Phylogenetic signals of size and shape data were estimated (by assessing the Blomberg’s K and K_mult_ metrics, respectively^79,80^) in order to decide if analyses should have been performed without the implementation of any phylogenetic correction (i.e., ordinary least squares regressions—OLS) or, alternatively, by applying phylogenetic comparative methods implementing two different models of evolution (i.e., Brownian Motion conditions—BM; the most likely model of evolution according to a phylogenetic ridge regression—RR) into phylogenetic generalized least squares (PGLS) regressions. Brownian motion evolution is a constant and non-directional random diffusion-like process as resulting from neutral evolution^81^. By contrast, phylogenetic ridge regression estimates branch-specific evolutionary rates and ancestral states under a wide range of models of trait evolution^82,83^ and multiplies branch lengths by branch-specific evolutionary rates to accommodate rate variation across the tree. All comparative analyses were performed twice, relying on two different phylogenies inclusive of fossil and living felids (taken from Piras et al.^64^ and Faurby et al.^84^) as estimates of evolutionary relationships. The best fitting regression model comparing different modes of evolution (i.e., BM versus RR PGLS relying on the same clade, phylogenetic tree, and landmark configuration) was estimated computing AIC scores. The impact of phylogenetic uncertainty (e.g., Piras et al.^64^ versus Faurby et al.^84^ tree) and landmark configuration (e.g., 30L versus 10L configuration) on CREA in felids was also assessed by comparing the results emerging from allometric regressions.

Additional regressions were performed using RCH as a predictor (instead of the natural logarithm of centroid size), after having estimated even in this case its phylogenetic signal, in order to quantify the impact of this biomechanical metric on cranial shape evolution in felids. RCH (i.e., canine crown height divided by the distance between postglenoid process of the jaw joint to the center of the canine) has been suggested to be a fundamental proxy to explain patterns of morphological variation within this family, particularly in sabertoothed cats, and it highly correlates with the degree of mouth opening (i.e., gape angle) in predatory species^63,85^. RCH scores were obtained from linear measures digitally collected (by a single operator – DT) on the cranial sample using the software MeshLab (version 2021.05).

All the regressions were performed using the R packages *geomorph*^86^ and *RRphylo*^82^. The significance of each test was assessed by performing 1000 simulations against random expectations. A multiple testing correction, known as Benjamini–Hochberg procedure, was applied to take into account the simultaneous implementation of several tests, which could inflate type I errors^87^. The morphological data, R code, and phylogenies used in this study are provided inSI Appendix. The visualization of opposite extremes of all the allometric and shape versus relative canine height trajectories was done warping, by means of thin-plate spline, a single specimen from our sample selected on a case-by-case basis.

### Graphical visualizations of multivariate trait spaces

In keeping with Mitteroecker et al.^88^ and Cardini and Polly^89^, the natural logarithm of centroid size was appended to the matrix of Procrustes shape coordinates to perform a principal component analysis (PCA) in the form space. Scatterplots of the first PCs in the form space can be used to quantitatively describe the main aspects of morphological change and explore differences and similarities across groups (in our case subfamilies), allowing us to graphically evaluate differences in the spatial orientation of their evolutionary trajectories, which are a proxy for the degree of divergence in their pooled within group allometric patterns^88^. This procedure was also repeated using RCH scores, instead of the natural logarithm of centroid size, in order to graphically visualize the evolutionary trajectories followed by each subfamily in the multivariate trait space describing both their shape (i.e., Procrustes coordinates) and biomechanical performance (i.e., RCH). All these analyses were performed on the 30L configuration and then repeated using the 10L configuration.

## Results

### Patterns of allometric shape changes

Phylogenetic signals of size and shape data were statistically significant concerning both the phylogenetic trees used in our analyses (i.e., Blomberg’s K = 1.580 and K_mult_ = 0.644 concerning Faurby et al.^84^ phylogeny, respectively; Blomberg’s K = 1.566 and K_mult_ = 0.653 concerning Piras et al.^64^ phylogeny, respectively, with all four tests showing P-values = 0.001), suggesting us to adopt phylogenetically-informed allometric regressions.

**Figure 2:**
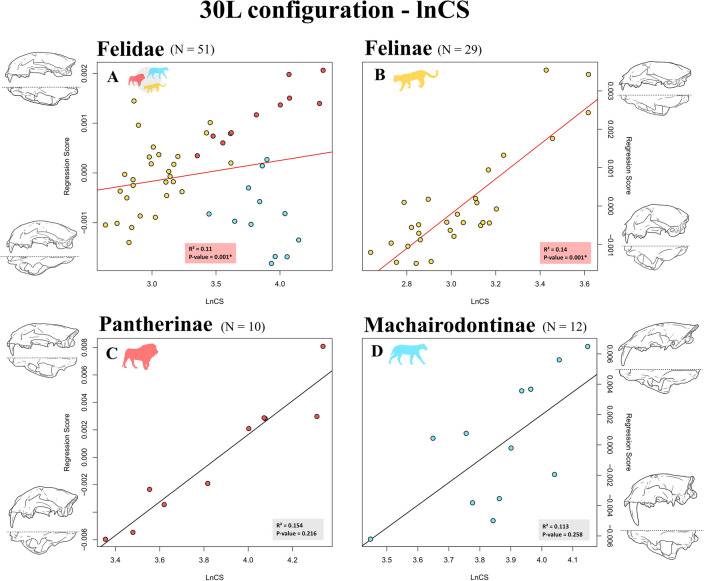
Scatterplots of shape regression scores versus natural logarithm of centroid size obtained using the 30L configuration, Faurby et al.^84^ phylogeny, and Brownian motion (BM) PGLS concerning Felidae (**A**), Felinae (**B**), Pantherinae (**C**), Machairodontinae (**D**). Craniofacial evolutionary allometry (CREA) is supported at the family level (**A**), but this pattern appears to be the product of a different impact of CREA on Felinae (i.e., strong impact—**B**), Pantherinae (i.e., weak impact—**C**), and Machairodontinae (i.e., no impact—**D**). Patterns of allometric shape variation are shown by means of 3D surfaces warped using thin-plate spline.

R^2^ ranged from 0.103 to 0.151 in all PGLS allometric regressions performed at the family level (Table 1). All P-values concerning these tests reached statistical significance, even after applying a Benjamini–Hochberg procedure (P-values = 0.001). RR PGLS regressions were the best fitting models according to the resulting AIC scores. Larger felids showed relatively enlarged rostra combined with reduced and arrow-shaped braincases (Fig. 2A for 30L configuration; Fig. S2A for 10L configuration). Larger species also experienced a dorso-ventral compression of the cranium.

**Table 1:** Results of the allometric regressions performed on 30L and 10L configurations using Brownian motion (BM) or phylogenetic ridge regression (RR) PGLS.

Sample	Phylogeny	Phylogenetic comparative method (PCM)	30L configuration					10L configuration				
			R^2^	F	Z	P-value	AIC	R^2^	F	Z	P-value	AIC
Felidae	Piras et al. 2018	BM PGLS	0.103	5.599	4.464	*0.001*	− 253.845	0.110	6.082	3.917	*0.001*	−217.932
		**RR PGLS**	**0.134**	**7.556**	**5.285**	***0.001***	− **264.444**	**0.139**	**7.901**	**4.498**	***0.001***	**− 236.081**
	Faurby et al. 2019	BM PGLS	0.110	6.069	4.621	*0.001*	− 251.932	0.105	5.745	3.617	*0.001*	− 206.300
		**RR PGLS**	**0.139**	**7.917**	**5.412**	***0.001***	**− 260.723**	**0.151**	**8.739**	**4.633**	***0.001***	**− 234.416**
Felinae	Piras et al. 2018	BM PGLS	0.143	4.523	3.687	*0.001*	− 169.789	0.176	5.757	3.531	*0.001*	− 151.349
		**RR PGLS**	**0.149**	**4.724**	**3.952**	***0.001***	**− 175.803**	**0.155**	**4.942**	**3.406**	***0.001***	**− 157.041**
	Faurby et al. 2019	BM PGLS	0.141	4.430	3.608	*0.001*	− 176.511	0.151	4.816	3.374	*0.001*	− 148.315
		**RR PGLS**	**0.155**	**4.942**	**4.154**	***0.001***	**− 179.503**	**0.161**	**5.177**	**3.597**	***0.001***	**− 158.062**
Pantherinae	Piras et al. 2018	BM PGLS	0.213	2.169	1.770	*0.026*	− 47.583	0.205	2.063	1.335	0.084	− 36.665
		**RR PGLS**	**0.221**	**2.266**	**1.936**	***0.019***	**− 51.377**	**0.207**	**2.089**	**1.413**	**0.082**	**− 41.016**
	Faurby et al. 2019	BM PGLS	0.154	1.452	0.863	0.216	− 40.523	0.107	0.959	0.233	0.430	− 29.153
		**RR PGLS**	**0.214**	**2.180**	**1.837**	***0.015***	**− 50.675**	**0.211**	**2.135**	**1.434**	**0.071**	**− 41.001**
Machairodontinae	Piras et al. 2018	BM PGLS	**0.083**	**0.903**	**− 0.087**	**0.537**	**− 48.210**	0.101	1.129	0.421	0.365	− 40.128
		RR PGLS	0.093	1.020	0.180	0.428	− 47.543	**0.112**	**1.263**	**0.661**	**0.265**	**− 40.496**
	Faurby et al. 2019	**BM PGLS**	**0.113**	**1.272**	**0.680**	**0.258**	**− 48.648**	**0.109**	**1.223**	**0.600**	**0.275**	**− 41.568**
		RR PGLS	0.226	2.919	2.276	*0.011*	− 46.464	0.200	2.502	2.016	*0.015*	− 41.039

Significant P-values at α = 0.05 are underlined, whereas P-values still significant after applying a Benjamini–Hochberg procedure are in Italics. Best fitting models according to the Akaike information criterion (AIC) are in bold.

Results of allometric regressions performed within the subfamily Felinae largely overlapped those obtained at the family level: R^2^ ranged from 0.141 to 0.176, and all P-values concerning these tests reached statistical significance, even after applying a Benjamini–Hochberg procedure (P-values = 0.001—Table 1). RR PGLS regressions were the best fitting models according to the resulting AIC scores. Larger feline species were characterized by a relative increase in prognathism, as well as a proportional reduction of braincase (Figs. 2B, S2B).

Within the subfamily Pantherinae, R^2^ ranged from 0.107 to 0.221 in all PGLS allometric regressions (Table 1). P-values reached statistical significance in three out of eight regressions within this group (i.e., all of them relative to the 30L configuration), even after applying a Benjamini–Hochberg procedure. RR PGLS regressions were the best fitting models according to the resulting AIC scores. Allometric shape changes within Pantherinae described a dorso-ventral flattening and a relative reduction of the braincase in larger species, paired with a relative enlargement of both the rostrum and the nasal cavity (Figs. 2C, S2C).

Within Machairodontinae, R^2^ ranged from 0.083 to 0.226 in all PGLS allometric regressions (Table 1). Within this group, P-values were statistically significant only in RR PGLS regressions performed on both 30L and 10L configurations using the Faurby et al.^84^ phylogeny and kept their significance after applying a Benjamini–Hochberg procedure (P-values = 0.011 and 0.015, respectively). BM PGLS regressions were the best fitting models according to the resulting AIC scores, except for regressions performed on the 10L configuration using the Piras et al.^64^ phylogeny. Larger sabertoothed cats mainly differed from their smaller relatives by a marked widening of the sagittal region in the sagittal plane and a lateral compression of the cranium (Figs. 2D, S2D). Larger Machairodontinae showed a slight relative elongation of the rostrum in the sagittal plane, but, contrary to CREA expectations, they did not show a relative reduction of the braincase.

### Impact of landmark configuration and phylogeny on CREA

Both landmark configurations used in this study returned comparable results in terms of allometric shape variation. Changes in R^2^ produced by using a different landmark configuration were smaller than 0.048 for all the considered cases, regardless of the taxonomic group, comparative method or phylogeny (Table 1). 30L and 10L configurations returned comparable results in terms of statistical significance, except for three cases that reached statistical significance only relatively to the 30L configuration within the subfamily Pantherinae.

Changing the phylogenetic tree used as a background for the phylogenetic comparative methods had a negligible impact on CREA in felids and every other considered subclade. Variations in R^2^ produced by using different phylogenetic trees were smaller than 0.134 for all the considered cases. All PGLS regressions returned a similar statistical significance when comparing results obtained using the two phylogenies, except for two cases relative to the 30L configuration and one case relative to the 10L one (Table 1).

### Shape versus relative canine height regressions

Coherently with phylogenetic signal in shape data as detailed above, also RCH showed a significant Blomberg’s K relatively to both the adopted phylogenies (Blomberg’s K = 2.188 and 2.279 concerning Faurby et al.^84^ and Piras et al.^64^ phylogenies, respectively; P-values = 0.001), suggesting us to adopt phylogenetically-informed tests even for shape versus relative canine height regressions.

**Figure 3:**
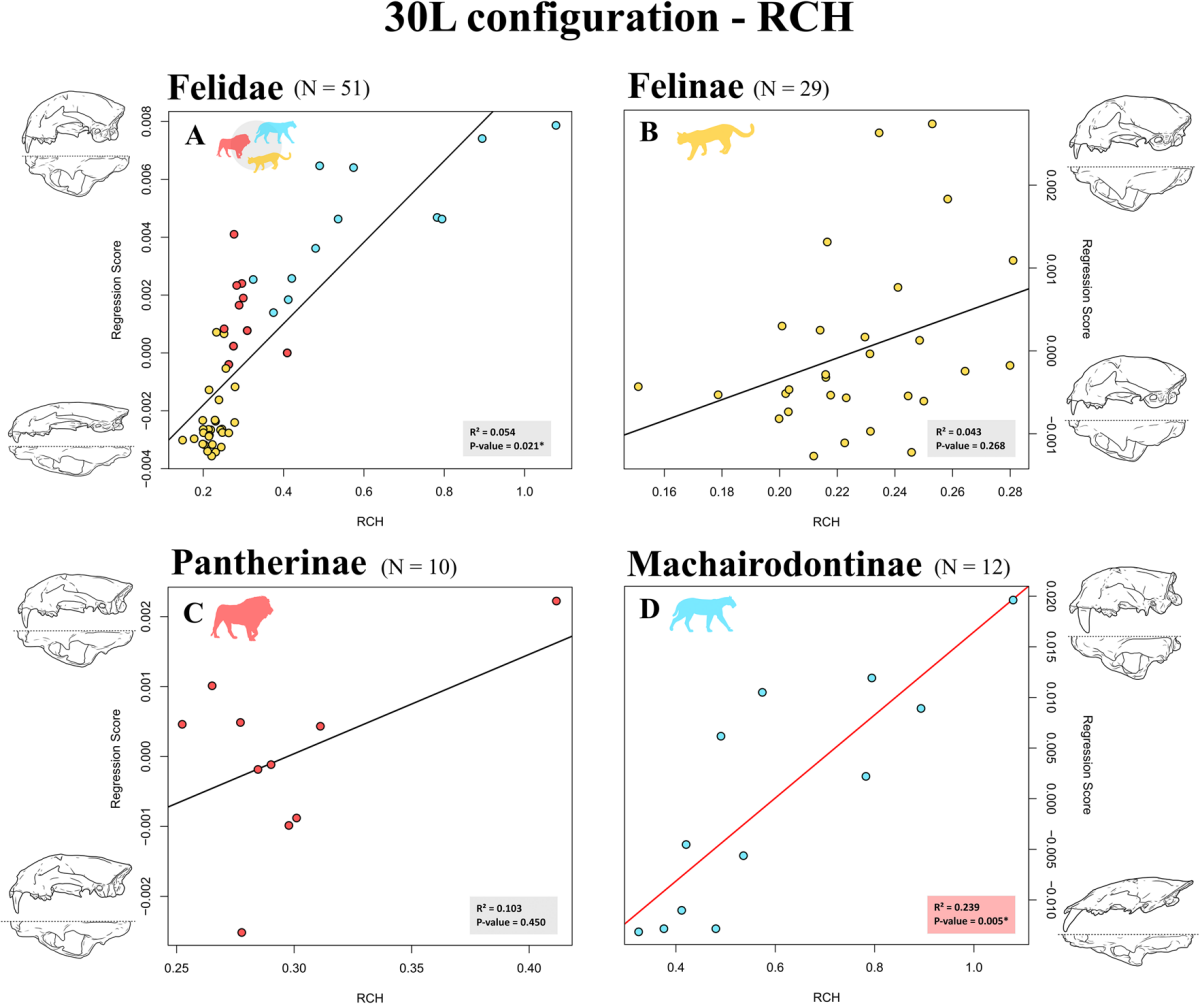
Scatterplots of shape regression scores versus relative canine height (RCH) obtained using the 30L configuration, Faurby et al.^84^ phylogeny, and Brownian motion (BM) PGLS concerning Felidae (**A**), Felinae (**B**), Pantherinae (**C**), Machairodontinae (**D**). Cranial shape is influenced by relative canine height at the family level (**A**), but this pattern appears to be the product of a different impact of relative canine height on Felinae (i.e., weak impact—**B**), Pantherinae (i.e., no impact—**C**), and Machairodontinae (i.e., strong impact—**D**). Patterns of shape variation are shown by means of 3D surfaces warped using thin-plate spline.

R^2^ ranged from 0.051 to 0.091 for all the shape versus relative canine height PGLS regressions performed at the family level (Table 2). All P-values reached statistical significance (P-values < 0.032) in this sample and seven out of eight of these P-values kept their statistical significance after applying a Benjamini–Hochberg procedure. RR PGLS regressions were the best fitting models according to the resulting AIC scores. Shape changes related to RCH at the family level followed a pattern in which species with relatively longer canines were characterised by a dorso-ventral expansion of the cranium, paired with an antero-posterior compression of this structure (Fig. 3A for 30L configuration; Fig. S3A for 10L configuration).

**Table 2:** Results of the shape versus relative canine height regressions performed on 30L and 10L configurations using Brownian motion (BM) or phylogenetic ridge regression (RR) PGLS.

Sample	Phylogeny	Phylogenetic Comparative Method (PCM)	30L configuration					10L configuration				
			R^2^	F	Z	P-value	AIC	R^2^	F	Z	P-value	AIC
Felidae	Piras et al. 2018	BM PGLS	0.051	2.635	2.086	*0.005*	− 229.274	0.061	3.170	2.142	*0.010*	− 208.431
		**RR PGLS**	**0.057**	**2.984**	**2.664**	***0.001***	**− 234.951**	**0.069**	**3.652**	**2.767**	***0.001***	**− 222.277**
	Faurby et al. 2019	BM PGLS	0.054	2.794	2.096	*0.021*	− 218.593	0.053	2.728	1.901	0.031	− 191.761
		**RR PGLS**	**0.077**	**4.089**	**3.399**	***0.001***	**− 234.910**	**0.091**	**4.886**	**3.355**	***0.001***	**− 222.456**
Felinae	Piras et al. 2018	BM PGLS	0.073	2.127	1.765	0.037	− 149.958	0.102	3.060	2.196	*0.009*	− 139.315
		**RR PGLS**	**0.072**	**2.100**	**1.946**	**0.031**	**− 152.940**	**0.058**	**1.666**	**1.228**	**0.112**	**− 143.702**
	Faurby et al. 2019	BM PGLS	0.043	1.206	0.607	0.268	− 149.676	0.044	1.228	0.601	0.273	− 133.711
		**RR PGLS**	**0.072**	**2.106**	**1.983**	***0.020***	**− 152.533**	**0.062**	**1.785**	**1.365**	**0.088**	**− 142.244**
Pantherinae	Piras et al. 2018	BM PGLS	0.148	1.393	0.743	0.255	− 37.309	0.042	0.349	− 0.849	0.796	− 31.965
		**RR PGLS**	**0.145**	**1.353**	**0.709**	**0.237**	**− 39.622**	**0.073**	**0.630**	**− 0.291**	**0.632**	**− 35.868**
	Faurby et al. 2019	BM PGLS	0.103	0.923	0.134	0.450	− 34.406	0.024	0.197	− 1.216	0.884	− 26.909
		**RR PGLS**	**0.148**	**1.386**	**0.720**	**0.257**	**− 39.858**	**0.076**	**0.662**	**− 0.402**	**0.652**	**− 36.077**
Machairodontinae	Piras et al. 2018	BM PGLS	0.140	1.629	1.293	0.104	− 50.526	0.150	1.770	1.324	0.075	− 43.897
		**RR PGLS**	**0.134**	**1.543**	**1.195**	**0.121**	**− 51.311**	**0.139**	**1.616**	**1.207**	**0.106**	**− 45.721**
	Faurby et al. 2019	BM PGLS	0.239	3.140	2.336	*0.005*	− 47.511	0.238	3.127	2.142	*0.014*	− 43.710
		**RR PGLS**	**0.207**	**2.613**	**2.292**	***0.005***	**− 48.670**	**0.182**	**2.218**	**1.899**	***0.018***	**− 45.277**

Significant P-values at α = 0.05 are underlined, whereas P-values still significant after applying a Benjamini–Hochberg procedure are in Italics. Best fitting models according to the Akaike information criterion (AIC) are in bold.

Cranial shape in felines was scarcely influenced by relative canine height since R^2^ ranged from 0.043 to 0.102 relatively to the PGLS regressions performed within this group, with four out of eight significant P-values and only two of them that reached statistical significance after applying a Benjamini–Hochberg procedure (Table 2). RR PGLS regressions were the best fitting models according to the resulting AIC scores. Shape changes related to RCH were negligible in felines and mainly consisted in a slight antero-posterior compression of the cranium in species with relatively longer canines (Figs. 3B, S3B).

Similar to felines, RCH had a weak impact on cranial shape in pantherines. R^2^ ranged from 0.024 to 0.148 for all the shape versus relative canine height PGLS regressions performed within Pantherinae (Table 2). None of the P-values reached statistical significance (P-values > 0.236) within this group. RR PGLS regressions were the best fitting models according to the resulting AIC scores. Shape changes related to RCH were negligible in pantherines and mainly occurred in the frontal region of the cranium (e.g., species with relatively longer canines had their zygomatic processes in a more anterior position than their counterparts with relatively smaller canines—Figs. 3C, S3C).

R^2^ ranged from 0.134 to 0.239 for all the shape versus relative canine height PGLS regressions performed within Machairodontinae (Table 2). Sabertoothed cats returned significant P-values in four out of eight regressions (i.e., all tests except for those relying on Piras et al.^64^ phylogeny), even after applying a Benjamini–Hochberg procedure. RR PGLS regressions were the best fitting models according to the resulting AIC scores. Shape changes related to RCH within sabertoothed cats followed a pattern in which species with higher RCH scores were characterised by a dorso-ventral expansion of the rostral region of the cranium, a reduced sagittal region, and occipital condyles occurring in a more posterior position than their counterparts with relatively smaller canines (Figs. 3D, S3D).

### Graphical visualizations of multivariate trait spaces

**Figure 4:**
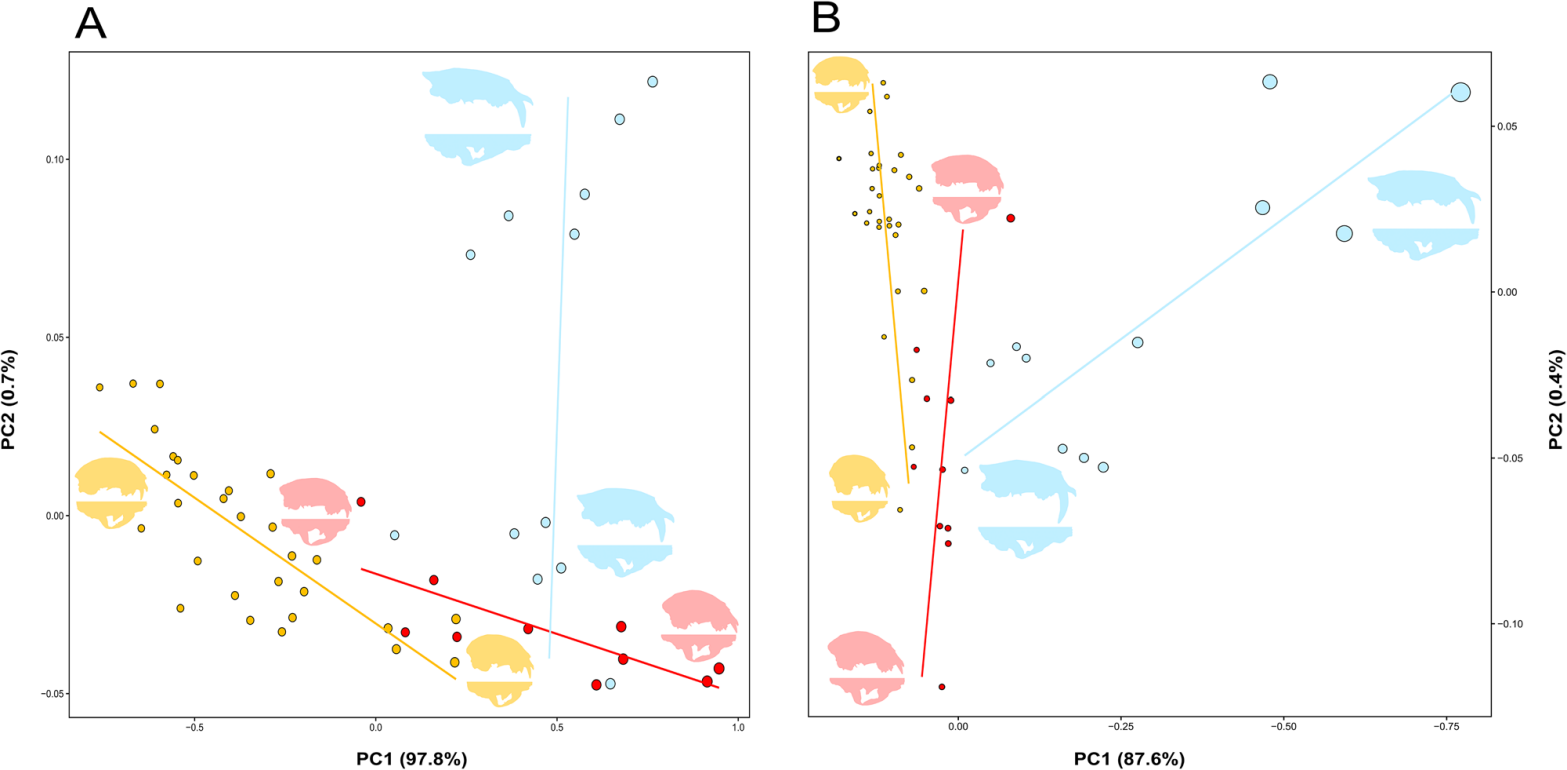
Scatterplots of felid cranial variation (relative to the 30L configuration) in the form (i.e., shape + size) space (**A**) and in the multivariate trait space obtained combining shape and relative canine height variables (**B**) summarized by PC1 (97.8% and 87.6% of variance explained, respectively) and PC2 (0.7% and 0.4% of variance explained, respectively). Evolutionary trajectories relative to each subfamily of Felidae described in these multivariate trait spaces are represented by straight lines (Felinae in yellow, Pantherinae in red, and Machairodontinae in light blue). The size of the points is proportional to the natural logarithm of centroid size (**A**) or the relative canine height (**B**) of each species. Sabertoothed cats follow evolutionary trajectories that largely diverge from those shown by their conical-toothed counterparts in both the considered multivariate trait spaces. Patterns of cranial variation at the extreme ends of the evolutionary trajectories are shown by means of 3D surfaces warped using thin-plate spline.

PC1-PC2 form space visualizations highlighted a wide divergence between the evolutionary trajectory that characterise sabertoothed cats and those of conical-toothed cats, with a spatial orientation that was divergent from the trajectories of Felinae and Pantherinae (Fig. 4A for 30L configuration; Figure S4A for 10L configuration). A similar pattern emerged from PC1 to PC2 visualizations relative to the multivariate trait space obtained combining shape and relative canine height variables, which showed how evolutionary trajectory of Machairodontinae greatly differs from those of conical-toothed cats even in this multivariate trait space (Fig. 4B for 30L configuration; Fig. S4B for 10L configuration).

## Discussion

### Implications for the cranial evolution of felids

Assessing the strength and pervasiveness of evolutionary trends, such as CREA, is essential to shed light on macroevolutionary dynamics and helps clarifying whether developmental changes and biomechanical demands may alter expected patterns of cranial evolution over long time-scales. Felids epitomize a meaningful case study for research on evolutionary allometry in mammals due to their specialised cranial morphology, paired with the hypercarnivorous diet shared by the entire clade. They also represent a case study for clades that are putatively affected by ratchet-like mechanisms in morphological evolution (i.e., constant and directional evolution occurring in specific clades due to their impossibility to retreat back along their evolutionary trajectory and leading them to extreme and suboptimal trait values, by analogy with the so-called “Muller’s ratchet” mechanism described in molecular evolution^90,91^, as suggested for several groups of hypercarnivorous mammals, non-mammalian synapsids, and dinosaurs^58,92,93^. Our results provided support to the validity of CREA at the family level. However, this pattern is not conserved at smaller taxonomic scale as our results show that CREA pattern is strongly supported within the small and medium-sized felines, whereas big cats, like Pantherinae and Machairodontinae, conform weakly if not at all with CREA predictions. Overall, the adoption of different landmark configurations and phylogenetic hypotheses had a very limited impact on CREA pattern recognition within felids. RCH impacts cranial shape evolution at the family level, but this is largely due to the strong influence of this factor on sabertoothed cats (that do not support CREA at all). Cranial shape of conical-toothed cats is not impacted by RCH.

Conical-toothed cats returned different results in terms of presence and strength of CREA when comparing Felinae to Pantherinae. CREA was always significant within fossil and extant felines, basically reconfirming the results obtained in previous 2D GMM research performed on living cats^50^. Our results concerning felines are in line with the allometric patterns emerging from other clades of placentals and marsupial in which CREA was previously confirmed (e.g., wallabies and kangaroos, mongooses^14,89^) and the magnitude of cranial variation explained by evolutionary allometry in this group is comparable with the amount of cranial shape variation due to deep differences in dietary habits and terrestrial*/*aquatic lifestyle within carnivorans^94^. Consistently with our findings, Slater and Van Valkenburgh^85^ stated that shape evolution in the cranium of felines is mainly explained by allometric variations, rather than shape changes due to functional or phylogenetic factors. Even in the case of pantherines, we assessed the presence of CREA following the suggestions of Tamagnini et al.^50^ by analysing the fossil record of the group and relying on phylogenetic corrections adequate for handling extinct species. Our results partially differed from the findings of Tamagnini et al.^50^, who hypothesised the presence of CREA in modern pantherines, except for the genus *Neofelis*. Unexpectedly, our findings validated the occurrence of this evolutionary trend in Pantherinae only when several anatomical features were simultaneously taken into account (i.e., 30L configuration), but provided weak support when the focus was shifted more specifically on the relative proportions of the rostrum and the braincase (i.e., 10L configuration—Table 1). Our results supported the occurrence of overlapping trajectories of Felinae and Pantherinae in RCH + Shape morphospaces, which are consistent with previous research suggesting the existence of a single functional rule in conical-toothed felids^95,96^.

In keeping with our findings, previous GMM studies performed on 2D datasets led the authors to hypothesise a reduced impact of evolutionary allometry in determining cranial shape variations in many sabertoothed lineages (i.e., Machairodontinae, Barbourofelidae, and Nimravidae^63,85^. In particular, Meloro and Slater^63^ questioned the presence of a common cranial allometric pattern in sabertoothed cats. However, analyses performed by Meloro and Slater^63^ relied on phylogenetic comparative methods that accounted only for a Brownian Motion mode of evolution (i.e., potentially returning highly incorrect estimations in case of non-Brownian evolution occurring within the considered sample). Furthermore, that study used potentially problematic morphological data due to the potential issues resulting from the adoption of cranial lateral views in GMM^97,98^. Such criticalities were solved in our analytical framework and data by including multiple phylogenetic comparative methods that were also able to consider cases of non-Brownian evolution (e.g., RR PGLSs) and by studying shape matrices resulting from 3D GMM that were able to describe the spatial complexity of crania. Nevertheless, even if our analyses supported a different (but still statistically non-significant) allometric pattern in Machairodontinae, our results consistently support Meloro and Slater^63^ claiming that the adaptation to elongate upper canines “result in a decoupling of the allometry-driven feline pattern of integration between the rostrum and braincase”. This evidence also disproves the hypothesis that cranial shape evolution of sabertoothed cats results from a mere case of cooptation and extension of the allometric trend observed in conical-toothed cats^85^. The absence of a single allometric relationship within a group is a condition frequently reflected by the lack of a single functional optimum that derives from grouping species with different optimal functions, precluding the identification of a single trend^99^. This is in line with recent biomechanical simulations performed on several functional parameters (i.e., mandibular gape angle, bending strength, and bite force) that highlighted a remarkably high functional variability among different sabertoothed lineages (e.g., machairodontines and barbourofelids^100^). In particular, variations regarding gape angle, bite force, and bending strength values occurred at fast rates and sabertoothed lineages expanded into different regions of the multivariate trait space. Despite the occurrence of convergent evolution in the craniomandibular morphology of sabertooths^101^, this high functional variability disproved the existence of a single consistent trend towards functional optimization for sabertooth-like morphologies, probably as a consequence of slight differences in hunting/killing strategies resulting in several episodes of ecological niche partitioning within sabertoothed lineages^100,102–104^ (see also Chatar et al.^105^ for similar considerations concerning mandibular biomechanics).

Overall, our results confirmed the importance of RCH in cranial evolution of sabertoothed cats (except for the tests that relied on a specific phylogenetic hypothesis that assumed *Machairodus aphanistus* to belong to the tribe Machairodontini sensu Jiangzuo et al.^106^ and to be a sister species of the clade Homotherina—Piras et al.^64^) in keeping with Meloro and Slater^63^. The important role played by this biomechanical factor is likely to derive from the need for sabertooths to have a wide gape angle and to accommodate the enormous canine roots within their facial skeleton, that are cranial features that might have invalidated CREA within this group. By contrast, our analyses disproved the impact of RCH on cranial morphology of conical-toothed cats, a condition that might depend on the existence of strong biomechanical trade-offs between the need to increase gape and the ability to resist unpredictable loadings deriving from prey handling^62^.

### Potential biomechanical reasons and developmental pathways leading to the absence of CREA

Our understanding of a general pattern can be improved by explaining why exceptions occur. We unambiguously find sabertoothed cats to constitute a well-supported exception to CREA, while pantherines represent a borderline case for this evolutionary trend since the strength of evolutionary allometry within this group is generally weak. The members of both these clades can be considered snouted/massive headed cats^95^, characterised by elongated rostra that accommodate massive dentitions^107,108^, that is a condition that might have deep implications for their patterns of morphological evolution. In particular, the weakening of CREA within pantherines likely derives from their homogeneous adaptation for killing large preys, which is a feature known to exert strong evolutionary pressure on facial shortening (i.e., to enhance bite force) and masticatory muscle mass. Brain volume of felids instead scale with cranial length lower than expected by geometric principles^109^. The combination of these patterns supports an expected shrinking of the braincase region counterbalanced also by a relatively short face, which might preclude CREA to be identified in full within pantherines. Similar arguments might apply to sabertoothed cats, whose crania are additionally influenced by the necessity to generate a wide gape, hence the evolution of bigger masticatory muscles and more elastic fibers^110^. Andersson et al.^111^ also proposed that this design is likely to have resulted in a possible behavioural shift (from jaw-powered killing bite of pantherines to neck-powered shear-biting of machairodontines). Another factor constraining cranial shape in sabertoothed species, potentially invalidating CREA pattern throughout the evolutionary history of the clade, is represented by specific adaptations of taxa such as *Machairodus* and *Homotherium*, like the enlargement of the mastoid cranial region, to enhance neck-powered shear biting^112–114^.

The fundamental role of teeth and masticatory muscles in determining the absence or presence of CREA was already suggested by previous studies that highlighted how the need to house large hypsodont teeth in specific ungulate lineages (e.g., African antelopes and equids^15^) or possess relatively long palate and tooth rows (e.g., clouded leopards^50^) might have the potential to break this evolutionary trend. The decoupling between relative face length and body size was also pointed out in the evolution of hominins and is hypothesized to be linked with a reduced need for powerful masticatory muscles (paired with an expansion of brain dimensions) deriving from a gradual increase of manually or industrially processed food consumption throughout the history of the clade^14,115^.

From a developmental point of view, variations in the relative face length are suggested to be linked with changes in the incidence of pure repetitive sequences, promoting new variances by duplication/deletion as emerged from studies on living carnivorans^116,117^. Sears and colleagues^118^ hypothesised that the number of *Runx2* tandem repeats represents a flexible genetic mechanism to regulate the facial length in carnivorans and alter the common patterns of interspecific allometric variations by modifying the rate and duration of bone development. Even if genetic data from fossilized tissues of carnivorans are still extremely limited (but see Barnett et al.^119^), heterochronic changes in the development of the dentition in sabertoothed cats were validated by stable carbon and oxygen isotope ratio analyses, for instance, suggesting that *Smilodon fatalis* evolved its long sabertooths by combining the canine development strategies of lions and tigers, that are a quick growth rate and a long growth over time, respectively^120^. Furthermore, patterns of convergent evolution have been recently suggested to occur in the morphology of deciduous upper canines (and in their replacement processes) in sabertoothed lineages, with clades like Barbourofelidae in which a delayed eruption of their deciduous sabertooths has already been described^121,122^. As described by Cardini^15^, CREA pattern is most likely produced by size diversification co-opting a common ontogenetic trajectory within mammals. It is therefore possible that developmental differences arising alongside facial length reorganisation may have altered strength and trajectories of macroevolutionary cranial allometry within felids. This hypothesis seems to be confirmed by the results of Krone et al.^45^ suggesting that divergent ontogenetic trajectories might explain the lack of support for CREA in many nonmammalian synapsid clades. Therefore, it is plausible that the absence of a CREA pattern within Machairodontinae is a consequence of the interaction of functional and developmental factors, even if the latters remain to be explored in more detail.

### Evolutionary trends and morphodynamics in scenarios of morphological evolution impacted by ratchet-like mechanisms

All these considerations lead us to hypothesise that biomechanic adapations, as well as the underlying developmental factors behind them, are key elements in determining the presence and strength of CREA. This is unsurprising considering that recent research on phenotypic evolutionary trends pointed out how this type of evolutionary pattern is heavily influenced by a complex interplay between evolutionary factors such as environment, evo-devo constraints, phylogeny, and biological function (i.e., theory of morphodynamics^3,123,124^. The acquisition of extreme features concerning any of these evolutionary factors (e.g., adapting biomechanically demanding structures such as sabertoothed upper canines; occupying extremely narrow and specialised ecological niches) is likely to be associated with peculiar patterns of morphological evolution, determining potential exceptions to common biological rules such as CREA, as observed in many hyper-specialised extinct lineages of nonmammalian synapsids often characterised by higher extinction rates due to their vulnerability to sudden ecological changes^45^ (see also Piras et al.^64^ and Machado^125^ for analogous considerations in sabertoothed cats and canids, respectively). This vulnerability is possibly resulting from ratchet-like mechanisms of morphological evolution, that are known to force many hypercarnivorous clades to evolve gradually more and more extreme adaptations related to their predatory strategy^58,92^. The results emerging from our study are consistent with the findings of Lamsdell^126^, who hypothesised, by studying the body trait variations of horseshoe crabs in response to aquatic-terrestrial transitions, a strong role of heterochronic changes in shaping their morphological evolution in presence of ratchet-like mechanisms. In particular, heterochrony was suggested to act on phenotypes by producing sudden peramorphoclines and paedomorphoclines and altering the pre-existing allometric trajectories^126,127^. Coherently with these findings, our results suggest that species with the most extreme morphologies (i.e., sabertoothed cats, in our case) evolve such features by deeply altering common patterns of evolutionary integration, like the braincase/face covariation that underpins CREA pattern^128,129^. In this sense, further research on clades known for having escaped conditions of morphological ratchet-like evolution, like the therapsid lineage Therocephalia, are expected to provide new insights on dynamics that link heterochrony, integration, and phenotypic evolution in such scenarios^92^.

Further research on cranial allometric patterns, possibly relying on phylogenetic trees deriving from advanced techniques like Bayesian inferences (see Jiangzuo et al.^106^ for an example on sabertoothed cats), should be more focused on lineages (and their fossil records) that are peculiar in terms of their ecological, biomechanical or evo-devo adaptations, since this would help clarifying the developmental-genetic pathways and processes involved in morphological evolution, with lineages that underwent ratchet-like evolution representing ideal targets. These case studies have the potential to elucidate the dynamics underpinning patterns of allometry and integration and their link with heterochronic changes, and, in this context, detecting exceptions to the biological rules, like CREA, might help researchers to refine their inferences on the evolutionary factors involved in such scenarios.

### Supplementary Information

Supplementary Information 1.

Supplementary Figure S1.

Supplementary Figure S2.

Supplementary Figure S3.

Supplementary Figure S4.

Supplementary Table S1.

Supplementary Table S2.

Supplementary Table S3.

Supplementary Table S4.

Supplementary Table S5.

## Data Availability

Morphological datasets, phylogenetic trees, and R script supporting the results of the present manuscript are archived in Dryad and/or provided as Supporting Information.
