# Supplementary material for: Conical and sabertoothed cats as an exception to craniofacial evolutionary allometry
Source: Sci Rep. 2023 Aug 21;13:13571. doi: 10.1038/s41598-023-40677-6 (PMC10442348; doi:10.1038/s41598-023-40677-6)
Supplement: Supplementary file 1 — Supplementary Information 1. [file 41598_2023_40677_MOESM1_ESM.docx]

**The complexity of biological rules: the example of conical and sabertoothed cats as an exception to craniofacial evolutionary allometry – Supplementary Information**

Davide Tamagnini^1,2^*, Margot Michaud^3^, Carlo Meloro^4^, Pasquale Raia^5^, Leopoldo Soibelzon^6,7^, P. Sebastián Tambusso^8,9^, Luciano Varela^8,9^, Luigi Maiorano^1,2^

^1^Department of Biology and Biotechnologies “Charles Darwin”, University of Rome “La Sapienza”, Rome, Italy

^2^Museum of Zoology, Sapienza Museum Centre, University of Rome “La Sapienza”, Rome, Italy

^3^Evolution & Diversity Dynamics Lab, University of Liège, Liège, Belgium

^4^Research Centre in Evolutionary Anthropology and Palaeoecology, School of Biological and

Environmental Sciences, Liverpool John Moores University, Liverpool, UK

^5^Dipartimento di Scienze della Terra, dell’Ambiente e delle Risorse, University of Naples Federico II, Napoli, Italy

^6^División Paleontología Vertebrados, Museo de La Plata, Facultad de Ciencias Naturales y Museo, Universidad Nacional de La Plata. Paseo del Bosque s/n, 1900, La Plata, Argentina

^7^Consejo Nacional de Investigaciones Científicas y Tecnicas (CONICET), Godoy Cruz 2290 (C1425FQB), CABA, Argentina

^8^Departamento de Paleontología, Facultad de Ciencias, Universidad de la República, Iguá 4225, 11400 Montevideo, Uruguay

^9^Servicio Académico Universitario y Centro de Estudios Paleontológicos (SAUCE-P), Universidad de la República, Santa Isabel s/n, 91500 Sauce, Departamento de Canelones, Uruguay

***SI Appendix***

**Repeatability and precision of landmarks**

The repeatability and precision of landmark configurations (i.e., digitizing error) were evaluated considering each specimen included in the total sample. To do so, landmarks were digitized twice by the same operator (DT) with a ten-day interval in order to assess the digitising error (Viscosi and Cardini 2011). For both landmark configurations (i.e., 30L and 10L) adopted in this study, a cluster analysis relying on the unweighted pair group method with arithmetic mean (UPGMA) was performed on the Procrustes coordinates resulting from first and second replicas. In both the configurations, the two replicas of the same individual clustered together for almost every specimen (i.e., 97 out of 98 correct pairs in the 30L configuration and 94 out of 98 correct pairs in the 10L configuration representing 99% and 96% of total cases, respectively), showing that the shape components resulting from both these landmark configurations were highly repeatable. For size, a correlation between the centroid sizes of the replicas was computed for each configuration and both these tests returned a correlation coefficient (r) higher than 0.999 (P-value = 0.0001 in both cases), indicating that also the size component was highly repeatable.

**Sexual dimorphism**

The impact of sexual dimorphism in shape was assessed performing UPGMA cluster analyses on a subsample including the 21 living species of felids (55% of the existing species diversity) for which at least one male and one female per species were included in the total sample. In particular, a UPGMA cluster analysis was performed, for both landmark configurations, on the Procrustes coordinates (averaged within species) corresponding to the female-only, the male-only and the pooled-sex means. In both configurations, the three means of the same species clustered together in a large number of cases (i.e., 17 out of 21 correct triads in the 30L configuration and 15 out of 21 correct triads in the 10L configuration representing 81% and 71% of total cases, respectively), indicating a reduced impact of sexual dimorphism on shape. For size, all the possible correlations between the centroid sizes of the three means were computed for each configuration. All the performed tests returned a correlation coefficient (r) higher than 0.966 (P-value = 0.0001 in all the cases), indicating that also the impact of sexual dimorphism on size was negligible.

To further investigate the impact of sexual dimorphism on CREA (CRaniofacial Evolutionary Allometry), we performed a battery of regressions similar to the one used in the main analyses (i.e., Brownian motion (BM) PGLS and phylogenetic ridge regression (RR) PGLS regressions relying on two different phylogenies - see subsection ‘Allometric and shape versus relative canine height regressions’ in the Methods section), comparing the results obtained using the female-only, male-only, and pooled-sex subsamples that were already used for UPGMA cluster analyses on shape and centroid size correlations. All the regressions were significant, regardless of the chosen landmark configuration (i.e., 30L – Table S3; 10L – Table S4), even after applying a Benjamini-Hochberg procedure (i.e., all P-values < 0.003). The amount of shape variance explained by evolutionary allometry varied little throughout the entire battery of regressions. In particular, the R^2^ obtained from BM PGLS regressions ranged from 0.230 to 0.332, regardless of the considered subsample, landmark configuration, and phylogenetic tree. Finally, the R^2^ obtained from RR PGLS regressions ranged from 0.162 to 0.372, regardless of the adopted subsample, landmark configuration, and phylogeny. RR PGLS regressions always resulted to be the best fitting models, according to the resulting AIC scores, if comparing RR PGLS versus BM PGLS regressions concerning the same subsample, landmark configuration, and phylogeny, except for the regressions performed in the male subsample using the 10L configuration. Overall, these results indicated a negligible impact of sexual dimorphism on CREA in felids.

**Missing landmarks and retrodeformation**

The presence of partially damaged specimens induced an extremely reduced number of missing landmarks both in 30L (70 missing landmarks out of 2940 expected landmarks – 2.4% of the total) and in 10L (28 out of 980 – 2.9% of the total) configurations. The estimation of missing landmarks was performed in two mutually exclusive methods as detailed in Table S5. For bilateral landmarks (Tab. S5, in red), we performed a symmetrization relying on their bilateral counterparts whenever available (Gunz et al. 2009). Symmetrizations were performed using the function *fixLMmirror* embedded in the package *Morpho* (Schlager 2017). For landmarks placed on the midline and bilateral landmarks for which a bilateral counterpart was not available (Tab. S5, in black), we used the thin-plate spline to interpolate missing landmarks on a reference specimen, that was obtained from a set of specimens for which all landmarks were present (Gunz et al. 2009). This set of specimens was selected in a hierarchical way case by case, meaning that the interpolation was performed at the lowest taxonomic level in which enough specimens were available to complete the procedure (i.e., at least other two non-damaged individuals are required - Tab. S5). Thin-plate spline-based estimations were performed using the function *estimate.missing* embedded in the package *Morpho.* Whenever both the methods of missing landmark estimation were required in the same specimen, we first performed the symmetrisation step and then the thin-plate spline-based interpolation.

The fossil specimens included in the present study were generally well preserved, particularly in terms of deformation caused by taphonomic processes. However, in the very few individuals (i.e., two specimens belonging to *Machairodus aphanistus* and *Panthera gombaszoegensis*, respectively) in which minor damages occurred, we virtually restored them using the retrodeformation procedure described in Schlager et al. (2018). In particular, these specimens were retrodeformed using up to ten pairs of symmetrical landmarks and up to five bilateral sets of semi-landmarks homogeneously distributed along curves. This procedure was performed using the package *Morpho*.
