# Supplementary material for: Conical and sabertoothed cats as an exception to craniofacial evolutionary allometry
Source: Sci Rep. 2023 Aug 21;13:13571. doi: 10.1038/s41598-023-40677-6 (PMC10442348; doi:10.1038/s41598-023-40677-6)
Supplement: Supplementary file 2 — Supplementary Figure S1. [file 41598_2023_40677_MOESM2_ESM.pdf]

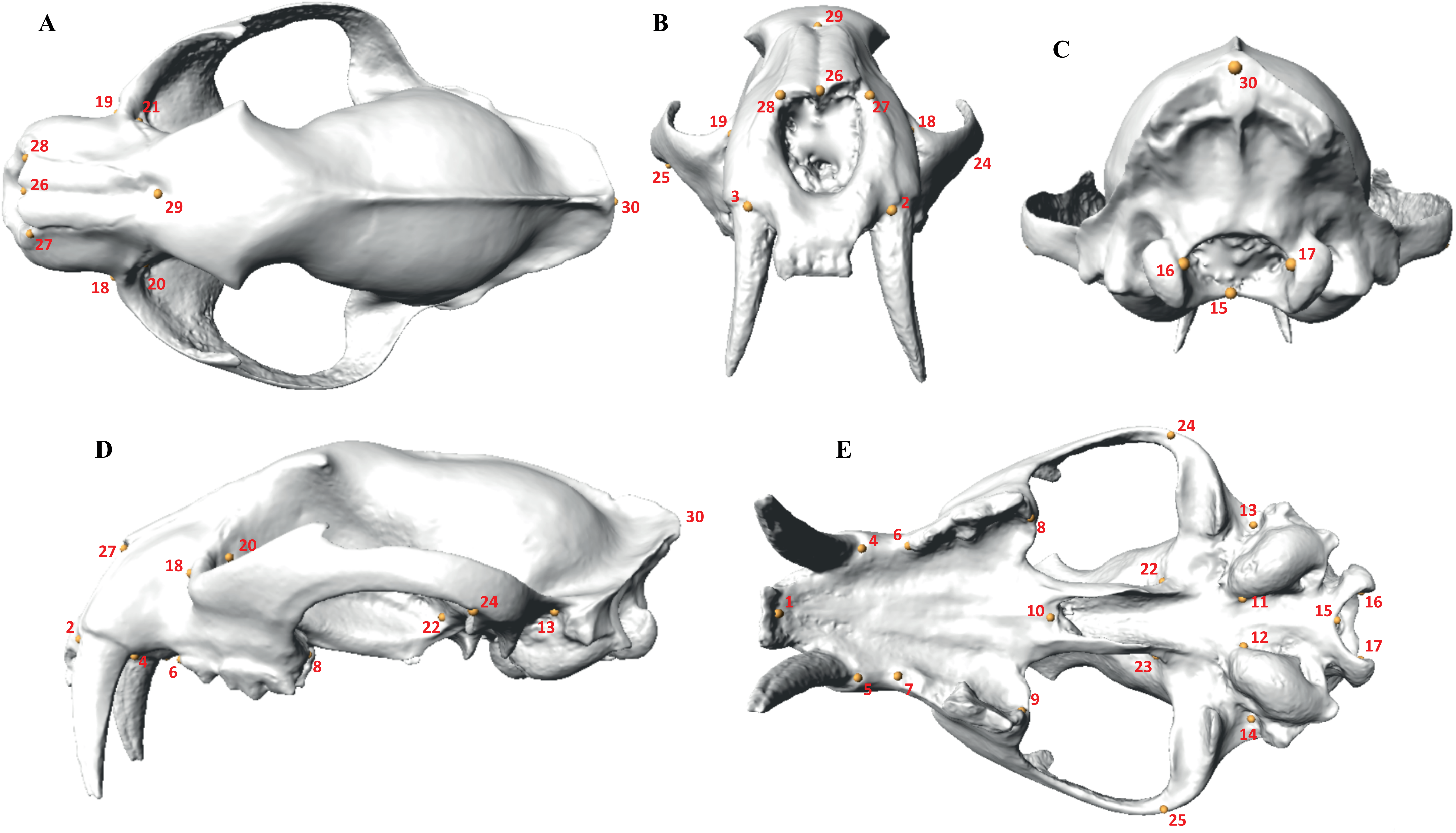

Figure S1: Landmarks used to quantify allometric shape variation in the cranium of felids. (A) dorsal view; (B) anterior view; (C) caudal view; (D) lateral view; (E) ventral view. Landmarks are represented by yellow dots.
