# Supplementary material for: Conical and sabertoothed cats as an exception to craniofacial evolutionary allometry
Source: Sci Rep. 2023 Aug 21;13:13571. doi: 10.1038/s41598-023-40677-6 (PMC10442348; doi:10.1038/s41598-023-40677-6)
Supplement: Supplementary file 3 — Supplementary Figure S2. [file 41598_2023_40677_MOESM3_ESM.pdf]

10L configuration - CS

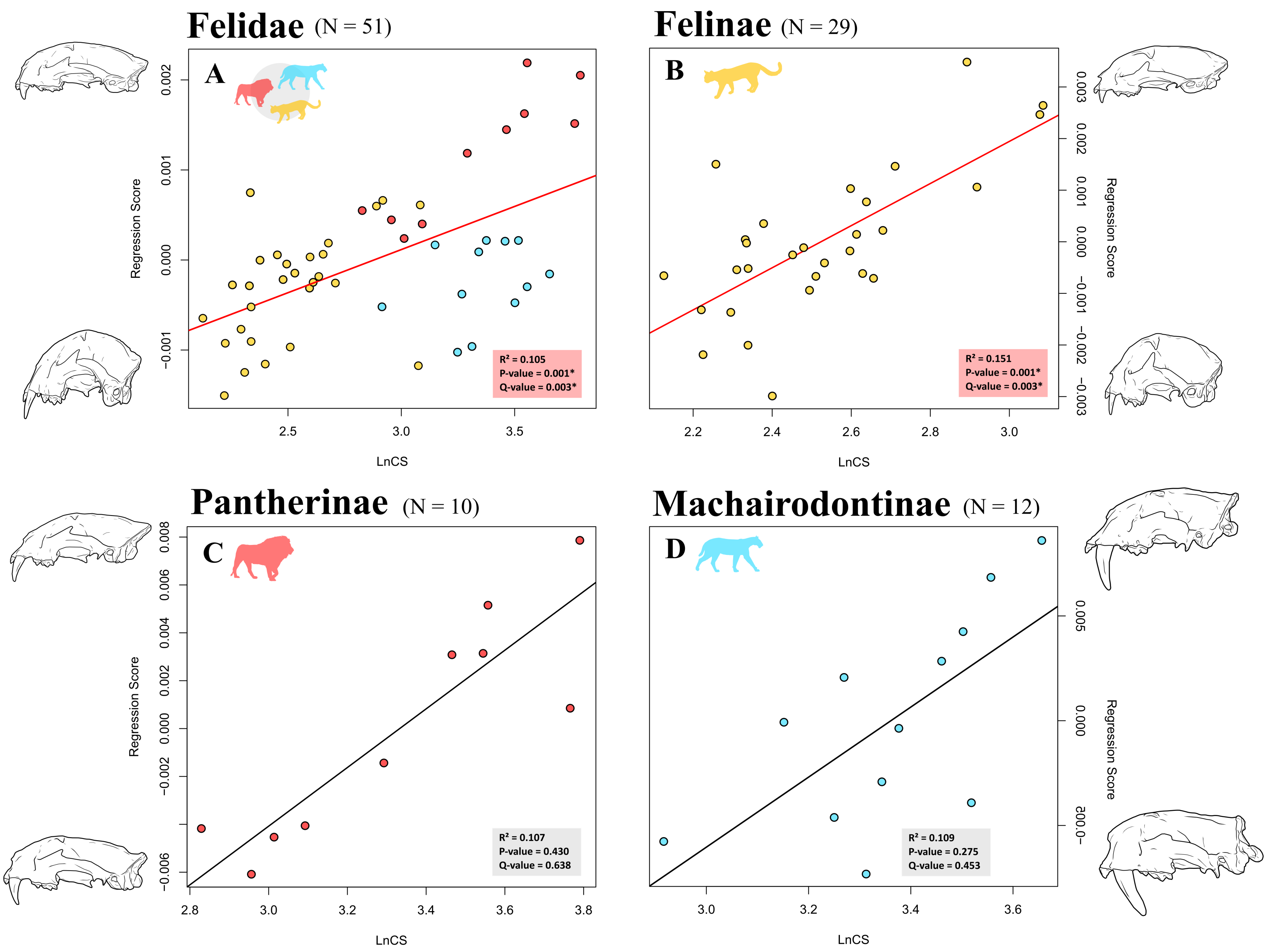

Figure S2: Scatterplots of shape regression scores versus natural logarithm of centroid size obtained using the 10L configuration, Faurby et al. (2019) phylogeny, and Brownian motion (BM) PGLS concerning Felidae (A), Felinae (B), Pantherinae (C), Machairodontinae (D). Craniofacial evolutionary allometry (CREA) is supported at the family level (A), but this pattern appears to be the product of a different impact of CREA on Felinae (i.e., strong impact – B), Pantherinae (i.e., weak impact – C), and Machairodontinae (i.e., no impact – D). Patterns of allometric shape variation are shown by means of 3D surfaces warped using thin-plate spline.
