# Supplementary material for: Conical and sabertoothed cats as an exception to craniofacial evolutionary allometry
Source: Sci Rep. 2023 Aug 21;13:13571. doi: 10.1038/s41598-023-40677-6 (PMC10442348; doi:10.1038/s41598-023-40677-6)
Supplement: Supplementary file 4 — Supplementary Figure S3. [file 41598_2023_40677_MOESM4_ESM.pdf]

10L configuration - RCH

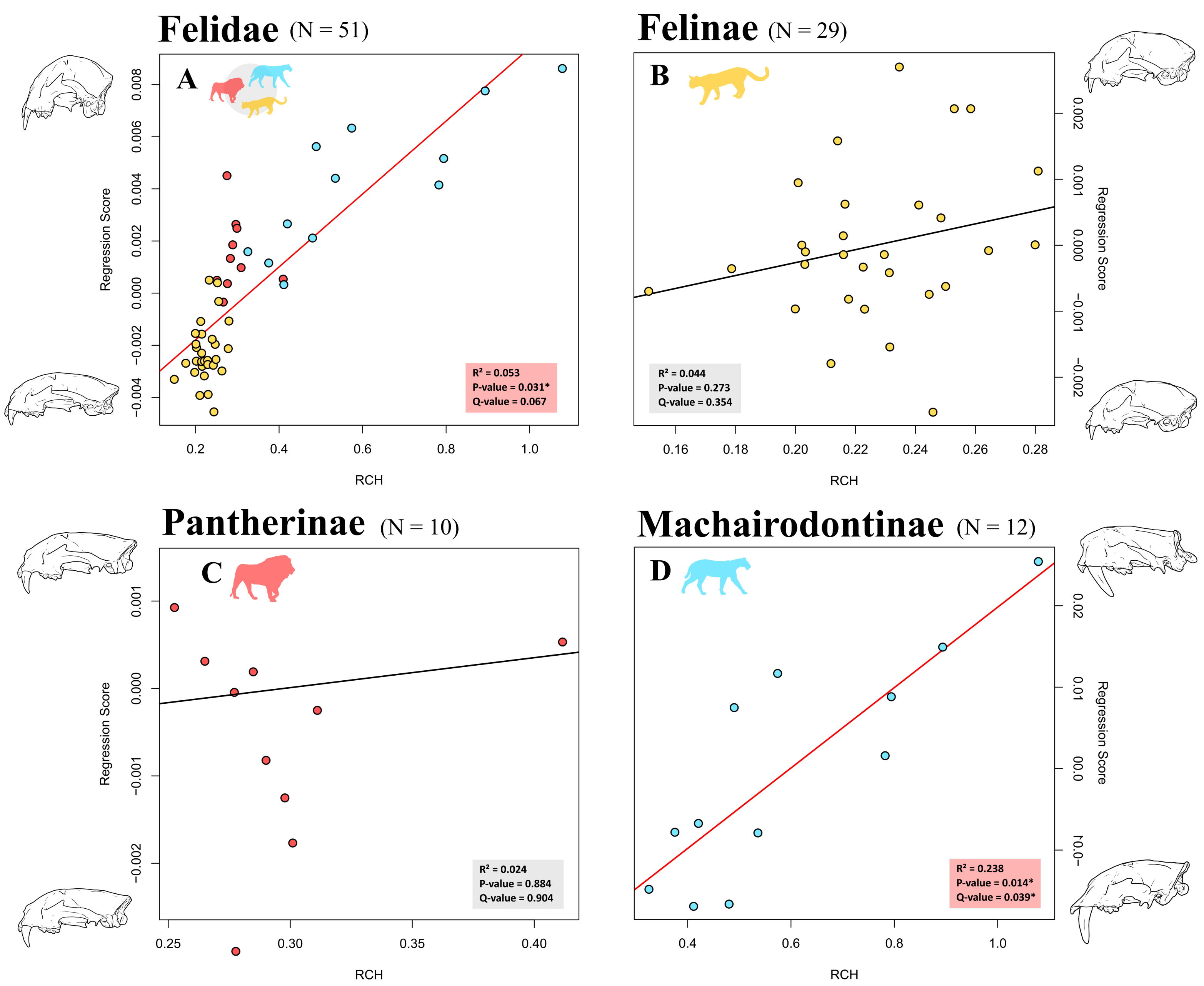

Figure S3: Scatterplots of shape regression scores versus relative canine height (RCH) obtained using the 10L configuration, Faurby et al. (2019) phylogeny, and Brownian motion (BM) PGLS concerning Felidae (A), Felinae (B), Pantherinae (C), Machairodontinae (D). Cranial shape is influenced by relative canine height at the family level (A), but this pattern appears to be the product of a different impact of relative canine height on Felinae (i.e., weak impact – B), Pantherinae (i.e., no impact – C), and Machairodontinae (i.e., strong impact – D). Patterns of shape variation are shown by means of 3D surfaces warped using thin-plate spline.
