# Supplementary material for: Conical and sabertoothed cats as an exception to craniofacial evolutionary allometry
Source: Sci Rep. 2023 Aug 21;13:13571. doi: 10.1038/s41598-023-40677-6 (PMC10442348; doi:10.1038/s41598-023-40677-6)
Supplement: Supplementary file 5 — Supplementary Figure S4. [file 41598_2023_40677_MOESM5_ESM.pdf]

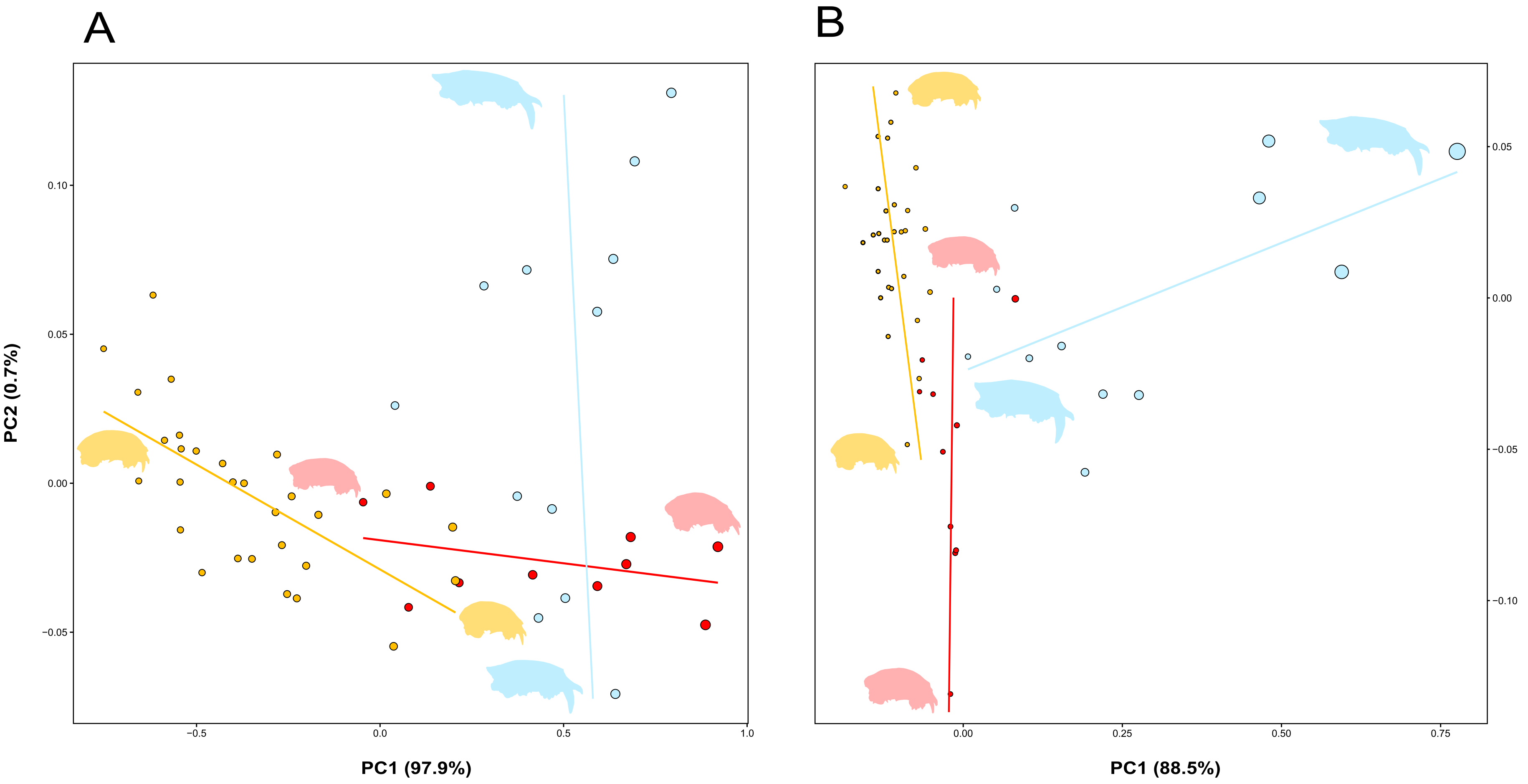

Figure S4: Scatterplots of felid cranial variation (relative to the 10L configuration) in the form (i.e., shape + size) space (A) and in the multivariate trait space obtained combining shape and relative canine height variables (B) summarized by PC1 (97.9% and 88.5% of variance explained, respectively) and PC2 (0.7% and 3.7% of variance explained, respectively). Evolutionary trajectories relative to each subfamily of Felidae described in these multivariate trait spaces are represented by straight lines (Felinae in yellow, Pantherinae in red, and Machairodontinae in light blue). The size of the points is proportional to the natural logarithm of centroid size (A) or the relative canine height (B) of each species. Sabertoothed cats follow evolutionary trajectories that largely diverge from those shown by their conical-toothed counterparts in both the considered multivariate trait spaces. Patterns of cranial variation at the extreme ends of the evolutionary trajectories are shown by means of 3D surfaces warped using thin-plate spline.
