# Supplementary material for: Conical and sabertoothed cats as an exception to craniofacial evolutionary allometry
Source: Sci Rep. 2023 Aug 21;13:13571. doi: 10.1038/s41598-023-40677-6 (PMC10442348; doi:10.1038/s41598-023-40677-6)
Supplement: Supplementary file 6 — Supplementary Table S1. [file 41598_2023_40677_MOESM6_ESM.pdf]

ID	Species name	Subfamily	Sex
Acinonyx_jubatus_F_MZUF-1831	*Acinonyx jubatus*	Felinae	Female
Acinonyx_jubatus_M_MZUF-2135	*Acinonyx jubatus*	Felinae	Male
Acinonyx_pardinensis_U_NMBS.St.V.975	*Acinonyx pardinensis*	Felinae	Unknown
Caracal_aurata_F_MNHN.CG1939.687	*Caracal aurata*	Felinae	Female
Caracal_aurata_M_MNHN.CG1940-1213	*Caracal aurata*	Felinae	Male
Caracal_caracal_F_MNHN.CG2015-2093	*Caracal caracal*	Felinae	Female
Caracal_caracal_M_MZUF-1752	*Caracal caracal*	Felinae	Male
Catopuma_temminckii_F_MNHN.CG1939.2152	*Catopuma temminckii*	Felinae	Female
Catopuma_temminckii_M_MNHN.CG1962-2927	*Catopuma temminckii*	Felinae	Male
Dinofelis_barlowi_U_DNMNH.BF55-22	*Dinofelis barlowi*	Machairodontinae	Unknown
Dinofelis_piveteaui_U_DNMNH.KA61	*Dinofelis piveteaui*	Machairodontinae	Unknown
Felis_bieti_U_MNHN.CG1893-151	*Felis bieti*	Felinae	Unknown
Felis_chaus_F_MNHN.CG2015-1302	*Felis chaus*	Felinae	Female
Felis_chaus_U_MZUF-12308	*Felis chaus*	Felinae	Unknown
Felis_concolor_U_PRIZ891	*Puma concolor*	Felinae	Unknown
Felis_margarita_U_IMNH.R-938	*Felis margarita*	Felinae	Unknown
Felis_serval_U_ac0141	*Leptailurus serval*	Felinae	Unknown
Felis_silverstris_F_MNHN.CG1995-448	*Felis silvestris*	Felinae	Female
Felis_silvestris_M_SAP.ZOO.84	*Felis silvestris*	Felinae	Male
Felis_silvestris_U_ha0066	*Felis silvestris*	Felinae	Unknown
Herpailurus_jaguaroundi_F_MNHN.CG2001-1292	*Herpailurus yagouaroundi*	Felinae	Female
Herpailurus_jaguaroundi_M_MNHN.CG1966-7	*Herpailurus yagouaroundi*	Felinae	Male
Homotherium_serum_U_TMM.933-3444	*Homotherium serum*	Machairodontinae	Unknown
Leopardus_colocolo_F_MNHN.CG1897-1261	*Leopardus colocolo*	Felinae	Female
Leopardus_geoffroyi_F_MNHN.CG1912-748	*Leopardus geoffroyi*	Felinae	Female
Leopardus_jacobita_U_MNHN.CG2006-546	*Leopardus jacobita*	Felinae	Unknown
Leopardus_pajeros_F_MLP.1913	*Leopardus colocolo*	Felinae	Female
Leopardus_pardalis_F_MNHN.CG1998-1866	*Leopardus pardalis*	Felinae	Female
Leopardus_pardalis_M_MNHN.CH1902-50	*Leopardus pardalis*	Felinae	Male
Leopardus_pardalis_U_SAP.ZOO.Aula_A	*Leopardus pardalis*	Felinae	Unknown
Leopardus_tigrina_F_MNHN.CG2006-542	*Leopardus tigrinus*	Felinae	Female
Leopardus_tigrinus_M_MZUF-4054	*Leopardus tigrinus*	Felinae	Male

Table S1: List of sampled specimens with ID code, species name, subfamily, sex, and museum location.

Leopardus_wiedii_F_IMNH.R-601	*Leopardus wiedii*	Felinae	Female
Leptailurus_serval_F_MNHN.CG1995-452	*Leptailurus serval*	Felinae	Female
Leptailurus_serval_M_MNHN.CG1958-164	*Leptailurus serval*	Felinae	Male
Lynx_canadensis_F_IMNH.R-213	*Lynx canadensis*	Felinae	Female
Lynx_canadensis_M_UWBM80612	*Lynx canadensis*	Felinae	Male
Lynx_issiodorensis_U_MNCN63887	*Lynx issiodorensis*	Felinae	Unknown
Lynx_issiodorensis_U_NMBS.Prr.200	*Lynx issiodorensis*	Felinae	Unknown
Lynx_lynx_F_MG-2-2013_852	*Lynx lynx*	Felinae	Female
Lynx_lynx_M_MG-2-2013_839	*Lynx lynx*	Felinae	Male
Lynx_pardina_U_MNCN16784	*Lynx pardinus*	Felinae	Unknown
Lynx_rufus_F_MNHN.CG2012-1024	*Lynx rufus*	Felinae	Female
Lynx_rufus_M_UV.155	*Lynx rufus*	Felinae	Male
Lynx_rufus_M_UWBM32046	*Lynx rufus*	Felinae	Male
Lynx_rufus_U_IMNH.R-115	*Lynx rufus*	Felinae	Unknown
Machairodus_aphanistus_U_BAT-105-E6-92	*Machairodus aphanistus*	Machairodontinae	Unknown
Machairodus_giganteus_U_HD-9196	*Amphimachairodus giganteus*	Machairodontinae	Unknown
Mayailurus_iriomotensis_M_PRIZ774	*Prionailurus bengalensis*	Felinae	Male
Megantereon_cultridens_U_NMBS.L.P.18	*Megantereon cultridens*	Machairodontinae	Unknown
Megantereon_cultridens_U_NMBS.Se.311	*Megantereon cultridens*	Machairodontinae	Unknown
Megantereon_nihowanensis_U_CB-20	*Megantereon nihowanensis*	Machairodontinae	Unknown
Neofelis_diardi_M_MNHN.CG1879-2133	*Neofelis diardi*	Pantherinae	Male
Neofelis_nebulosa_F_MNHN.CG1971-86	*Neofelis nebulosa*	Pantherinae	Female
Neofelis_nebulosa_M_MZUF-1024	*Neofelis nebulosa*	Pantherinae	Male
Otocolobus_manul_F_MNHN.CG2009.251	*Otocolobus manul*	Felinae	Female
Otocolobus_manul_M_MNHN.CG2010-646	*Otocolobus manul*	Felinae	Male
Panthera_atrox_U_CB2900-3	*Panthera atrox*	Pantherinae	Unknown
Panthera_gombaszoegensis_U_NMBS.V.A.1953	*Panthera gombaszoegensis*	Pantherinae	Unknown
Panthera_leo_F_MNHN.A12259	*Panthera leo*	Pantherinae	Female
Panthera_leo_M_MNHN.CG1938-632	*Panthera leo*	Pantherinae	Male
Panthera_leo_U_ab0030	*Panthera leo*	Pantherinae	Unknown
Panthera_leo_U_ab0031	*Panthera leo*	Pantherinae	Unknown
Panthera_leo_U_DUNUC2021	*Panthera leo*	Pantherinae	Unknown
Panthera_leo_U_MVZ.117849	*Panthera leo*	Pantherinae	Unknown

Panthera_leo_U_SAP.ZOO.Sala_lettura	*Panthera leo*	Pantherinae	Unknown
Panthera_onca_F_MNHN.CG1962-2880	*Panthera onca*	Pantherinae	Female
Panthera_onca_M_MZUF-501	*Panthera onca*	Pantherinae	Male
Panthera_onca_U_MZB2003-1528	*Panthera onca*	Pantherinae	Unknown
Panthera_onca_U_PRIZ890	*Panthera onca*	Pantherinae	Unknown
Panthera_onca_U_WML.D.2-1.11.1853	*Panthera onca*	Pantherinae	Unknown
Panthera_pardus_F_MZUF-1221	*Panthera pardus*	Pantherinae	Female
Panthera_pardus_M_MNHN.CG1998-1249	*Panthera pardus*	Pantherinae	Male
Panthera_pardus_U_AMNH.113745	*Panthera pardus*	Pantherinae	Unknown
Panthera_pardus_U_IMNH.R-2372	*Panthera pardus*	Pantherinae	Unknown
Panthera_pardus_U_WML.18.5.97.4	*Panthera pardus*	Pantherinae	Unknown
Panthera_spelaea_U_IMNH.###	*Panthera spelaea*	Pantherinae	Unknown
Panthera_tigris_F_MNHN.CG1895-355	*Panthera tigris*	Pantherinae	Female
Panthera_tigris_M_MNHN.CG1985-1860	*Panthera tigris*	Pantherinae	Male
Panthera_tigris_U_SAP.ANTRO.2954	*Panthera tigris*	Pantherinae	Unknown
Panthera_uncia_F_MNHN.CG2016-1664	*Panthera uncia*	Pantherinae	Female
Panthera_uncia_M_MNHN.CG1998-1248	*Panthera uncia*	Pantherinae	Male
Pardofelis_marmorata_U_MNHN.CG1886-25	*Pardofelis marmorata*	Felinae	Unknown
Prionailurus_bengalensis_F_MNHN.CG1954-293	*Prionailurus bengalensis*	Felinae	Female
Prionailurus_planiceps_U_MNHN.CG1873-228	*Prionailurus planiceps*	Felinae	Unknown
Prionailurus_rubiginosus_U_MNHN.CG1872-70	*Prionailurus rubiginosus*	Felinae	Unknown
Prionailurus_viverrinus_F_MNHN.CG2015-1300	*Prionailurus viverrinus*	Felinae	Female
Puma_concolor_F_UV.4117	*Puma concolor*	Felinae	Female
Puma_concolor_M_MNHN.CG1926-250	*Puma concolor*	Felinae	Male
Puma_concolor_U_IMNH.R-27	*Puma concolor*	Felinae	Unknown
Puma_concolor_U_ISM.ZOO.693928	*Puma concolor*	Felinae	Unknown
Puma_concolor_U_MZB2003-1534	*Puma concolor*	Felinae	Unknown
Smilodon_fatalis_U_F.AM.14349	*Smilodon fatalis*	Machairodontinae	Unknown
Smilodon_neogaeus_U_MSMN.V371	*Smilodon populator*	Machairodontinae	Unknown
Smilodon_populator_U_MNHN-P-957	*Smilodon populator*	Machairodontinae	Unknown
Therailurus_diastemata_U_NMBS.Rss83	*Dinofelis diastemata*	Machairodontinae	Unknown
Xenosmilus_hodsonae_U_BC-113	*Xenosmilus hodsonae*	Machairodontinae	Unknown
Yoshi_garevskii_U_MMNH-Sk-69	*Yoshi garevskii*	Machairodontinae	Unknown
