# Supplementary material for: Conical and sabertoothed cats as an exception to craniofacial evolutionary allometry
Source: Sci Rep. 2023 Aug 21;13:13571. doi: 10.1038/s41598-023-40677-6 (PMC10442348; doi:10.1038/s41598-023-40677-6)
Supplement: Supplementary file 7 — Supplementary Table S2. [file 41598_2023_40677_MOESM7_ESM.pdf]

Landmark	Description
1	Prosthion: antero-inferior point on projection of pre-maxilla between central incisors
2, 3	Antero-medial point on the alveolar margin of the canine
4, 5	Postero-medial point on the alveolar margin of the canine
6, 7	Maxilla: anterior extreme of tooth row (before first premolar)
8, 9	Maxilla: posterior midpoint onto alveolar margin of last molar
10	Palatine: posterior edge on the midline
11, 12	Basioccipital, basisphenoid and tympanic bulla: meeting point
13, 14	Dorsal tip of acoustic meatus
15	Basion: anterior-most point of foramen magnum
16, 17	Posterior extremity of occipital condyle along margin of foramen magnum
18, 19	Infraorbital foramen (dorsal tip on side external to the orbit)
20, 21	Lacrimal foramen
22, 23	Foramen rotundum
24, 25	Zygo-temp inferior: infero-lateral point of zygomatico (jugal) - temporal (squamosal) suture on lateral face of zygomatic arch
26	Rhinion: most anterior midline point on nasals
27, 28	Nasal And pre-maxilla: meeting point on margin of piriform aperture
29	Nasion: midline point on fronto-nasal suture
30	Inion: most posterior point of the cranium

Table S2: Definitions of the anatomical landmarks used in the 30L configuration. The 10L configuration includes only the rows with grey background.
