# Supplementary material for: Conical and sabertoothed cats as an exception to craniofacial evolutionary allometry
Source: Sci Rep. 2023 Aug 21;13:13571. doi: 10.1038/s41598-023-40677-6 (PMC10442348; doi:10.1038/s41598-023-40677-6)
Supplement: Supplementary file 8 — Supplementary Table S3. [file 41598_2023_40677_MOESM8_ESM.pdf]

Dataset	Sample	Phylogeny	Phylogenetic Comparative Method (PCM)	R^2	F	Z	P-value	AIC
Pooled-sex	Felidae	Piras et al. 2018	BM PGLS	0,321	9,002	3,854	<u>*0,001*</u>	-105,979
			**RR PGLS**	**0,259**	**6,641**	**3,978**	<u>***0,001***</u>	**-115,138**
		Faurby et al. 2019	BM PGLS	0,311	8,595	3,971	<u>*0,001*</u>	-112,403
			**RR PGLS**	**0,353**	**10,383**	**4,334**	<u>***0,001***</u>	**-116,237**
Female	Felidae	Piras et al. 2018	BM PGLS	0,230	5,680	3,697	<u>*0,001*</u>	-99,042
			**RR PGLS**	**0,185**	**4,326**	**3,788**	<u>***0,001***</u>	**-105,607**
		Faurby et al. 2019	BM PGLS	0,230	5,675	3,846	<u>*0,001*</u>	-103,460
			**RR PGLS**	**0,261**	**6,711**	**4,300**	<u>***0,001***</u>	**-106,178**
Male	Felidae	Piras et al. 2018	BM PGLS	0,275	7,203	3,934	<u>*0,001*</u>	-100,500
			**RR PGLS**	**0,202**	**4,820**	**3,680**	<u>***0,001***</u>	**-104,826**
		Faurby et al. 2019	BM PGLS	0,261	6,704	4,134	<u>*0,001*</u>	-105,487
			**RR PGLS**	**0,271**	**7,060**	**4,197**	<u>***0,001***</u>	**-107,269**

Table S3: Allometric regressions comparing pooled-sex, male and female datasets performed on the 30L configuration using Brownian Motion (BM) or phylogenetic ridge regression (RR) PGLS. Significant P-values at α = 0.05 are underlined, whereas P-values still significant after applying a Benjamini-Hochberg procedure are in Italics. Best fitting models according to the Akaike information criterion (AIC) are in bold.
