# Supplementary material for: Conical and sabertoothed cats as an exception to craniofacial evolutionary allometry
Source: Sci Rep. 2023 Aug 21;13:13571. doi: 10.1038/s41598-023-40677-6 (PMC10442348; doi:10.1038/s41598-023-40677-6)
Supplement: Supplementary file 9 — Supplementary Table S4. [file 41598_2023_40677_MOESM9_ESM.pdf]

Dataset	Sample	Phylogeny	Phylogenetic Comparative Method (PCM)	R^2	F	Z	P-value	AIC
Pooled-sex	Felidae	Piras et al. 2018	BM PGLS	0,332	9,455	3,823	*0,001*	-117,702
			**RR PGLS**	**0,275**	**7,213**	**3,756**	***0,001***	**-123,161**
		Faurby et al. 2019	BM PGLS	0,331	9,398	4,023	*0,001*	-122,937
			**RR PGLS**	**0,372**	**11,238**	**4,304**	***0,001***	**-125,713**
Female	Felidae	Piras et al. 2018	BM PGLS	0,239	5,954	3,540	*0,001*	-106,126
			**RR PGLS**	**0,195**	**4,614**	**3,457**	***0,001***	**-109,781**
		Faurby et al. 2019	BM PGLS	0,240	6,015	3,630	*0,001*	-107,716
			**RR PGLS**	**0,270**	**7,041**	**4,059**	***0,001***	**-109,968**
Male	Felidae	Piras et al. 2018	**BM PGLS**	**0,243**	**6,100**	**3,446**	***0,001***	**-99,304**
			RR PGLS	0,162	3,686	2,757	*0,002*	-97,081
		Faurby et al. 2019	**BM PGLS**	**0,239**	**5,978**	**3,655**	***0,001***	**-103,747**
			RR PGLS	0,256	6,554	3,778	*0,001*	-102,496

Table S4: Allometric regressions comparing pooled-sex, male and female datasets performed on the 10L configuration using Brownian Motion (BM) or phylogenetic ridge regression (RR) PGLS. Significant P-values at $\alpha = 0.05$ are underlined, whereas P-values still significant after applying a Benjamini-Hochberg procedure are in Italics. Best fitting models according to the Akaike information criterion (AIC) are in bold.
