# Supplementary material for: Conical and sabertoothed cats as an exception to craniofacial evolutionary allometry
Source: Sci Rep. 2023 Aug 21;13:13571. doi: 10.1038/s41598-023-40677-6 (PMC10442348; doi:10.1038/s41598-023-40677-6)
Supplement: Supplementary file 10 — Supplementary Table S5. [file 41598_2023_40677_MOESM10_ESM.pdf]

Number	ID	Missing Landmark (see Table)	Notes
1	Acinonyx_jubatus_F_MZUF-1831		
2	Acinonyx_jubatus_M_MZUF-2135	10	Estimated using all the other specimens belonging to the genus *Acinonyx* (except for Acinonyx_jubatus_U_ab0037)
3	Acinonyx_jubatus_U_ab0037		Escluded from every analysis because considered a case of species misidentification. It is not a specimen of *Acinonyx jubatus* as demonstrated in preliminary analyses (e.g., PCA)
4	Acinonyx_pardinensis_U_NMBS.St.V.975	13, 24, 28	Symmetrization
5	Caracal_aurata_F_MNHN.CG1939.687		
6	Caracal_aurata_M_MNHN.CG1940-1213	16	Symmetrization
7	Caracal_caracal_F_MNHN.CG2015-2093	26	Estimated using all the other specimens belonging to the genus *Caracal*
8	Caracal_caracal_M_MZUF-1752		
9	Catopuma_temminckii_F_MNHN.CG1939.2152		
10	Catopuma_temminckii_M_MNHN.CG1962-2927		
11	Dinofelis_barlowi_U_DNMNH.BF55-22	24, 25, 26	Estimated using all the other specimens belonging to the genus *Dinofelis*
12	Dinofelis_piveteaui_U_DNMNH.KA61	19, 22, 25	Symmetrization
13	Felis_bieti_U_MNHN.CG1893-151	15, 16, 17	Estimated using all the other specimens belonging to the genus *Felis*
14	Felis_chaus_F_MNHN.CG2015-1302		
15	Felis_chaus_U_MZUF-12308		
16	Felis_concolor_U_PRIZ891		
17	Felis_margarita_U_IMNH.R-938		
18	Felis_serval_U_ac0141		
19	Felis_silverstris_F_MNHN.CG1995-448		
20	Felis_silvestris_M_SAP.ZOO.84		
21	Felis_silvestris_U_ha0066		
22	Herpailurus_jaguaroundi_F_MNHN.CG2001-1292		
23	Herpailurus_jaguaroundi_M_MNHN.CG1966-7		
24	Homotherium_serum_U_TMM.933-3444		
25	Leopardus_colocolo_F_MNHN.CG1897-1261		
26	Leopardus_geoffroyi_F_MNHN.CG1912-748		
27	Leopardus_jacobita_U_MNHN.CG2006-546		
28	Leopardus_pajeros_F_MLP.1913		
29	Leopardus_pardalis_F_MNHN.CG1998-1866	14, 17	Symmetrization
30	Leopardus_pardalis_M_MNHN.CH1902-50		
31	Leopardus_pardalis_U_SAP.ZOO.Aula_A		
32	Leopardus_tigrina_F_MNHN.CG2006-542		
33	Leopardus_tigrinus_M_MZUF-4054	8	Symmetrization
34	Leopardus_wiedii_F_IMNH.R-601		
35	Leptailurus_serval_F_MNHN.CG1995-452		
36	Leptailurus_serval_M_MNHN.CG1958-164		
37	Lynx_canadensis_F_IMNH.R-213		
38	Lynx_canadensis_M_UWBM80612		
39	Lynx_issiodorensis_U_MNCN63887	17, 22, 23	Symmetrization and then estimation using all the other specimens belonging to the genus *Lynx*
40	Lynx_issiodorensis_U_NMBS.Prr.200	20, 21, 26, 29, 30	Estimated using all the other specimens belonging to the genus *Lynx*
41	Lynx_lynx_F_MG-2-2013_852		
42	Lynx_lynx_M_MG-2-2013_839		
43	Lynx_pardina_U_MNCN16784		
44	Lynx_rufus_F_MNHN.CG2012-1024		
45	Lynx_rufus_M_UV.155		
46	Lynx_rufus_M_UWBM32046		
47	Lynx_rufus_U_IMNH.R-115		
48	Machairodus_aphanistus_U_BAT-105-E6-92	18, 26, 28, 29	Symmetrization and then estimation using all the other specimens belonging to the clade Machairodontinae
49	Machairodus_giganteus_U_HD-9196	26, 29	Estimated using all the other specimens belonging to the clade Machairodontinae
50	Mayailurus_iriomotensis_M_PRIZ774		
51	Megantereon_cultridens_U_NMBS.L.P.18	1, 11, 13, 16, 22, 24	Symmetrization and then estimation using all the other specimens belonging to the clade Machairodontinae
52	Megantereon_cultridens_U_NMBS.Se.311		
53	Megantereon_nihowanensis_U_CB-20		
54	Neofelis_diardi_M_MNHN.CG1879-2133		
55	Neofelis_nebulosa_F_MNHN.CG1971-86		
56	Neofelis_nebulosa_M_MZUF-1024	17	Symmetrization
57	Otocolobus_manul_F_MNHN.CG2009.251		
58	Otocolobus_manul_M_MNHN.CG2010-646		
59	Panthera_atrox_U_CB2900-3		
60	Panthera_gombaszoegensis_U_NMBS.V.A.1953	1, 3, 5, 10, 15, 16, 17, 24, 26, 27, 28, 30	Symmetrization and then estimation using all the other specimens belonging to the genus *Panthera*
61	Panthera_leo_F_MNHN.A12259	13	Symmetrization
62	Panthera_leo_M_MNHN.CG1938-632		
63	Panthera_leo_U_ab0030		
64	Panthera_leo_U_ab0031		
65	Panthera_leo_U_DUNUC2021	10	Estimated using all the other specimens belonging to the species *Panthera leo*
66	Panthera_leo_U_MVZ.117849		
67	Panthera_leo_U_SAP.ZOO.Sala_lettura	15, 16, 17	Estimated using all the other specimens belonging to the species *Panthera leo*
68	Panthera_onca_F_MNHN.CG1962-2880		
69	Panthera_onca_M_MZUF-501		
70	Panthera_onca_U_MZB2003-1528		
71	Panthera_onca_U_PRIZ890		
72	Panthera_onca_U_WML.D.2-1.11.1853		
73	Panthera_pardus_F_MZUF-1221	10, 12, 14	Symmetrization and then estimation using all the other specimens belonging to the species *Panthera pardus*
74	Panthera_pardus_M_MNHN.CG1998-1249		
75	Panthera_pardus_U_AMNH.113745		
76	Panthera_pardus_U_IMNH.R-2372	3, 5, 7	Symmetrization
77	Panthera_pardus_U_WML.18.5.97.4		
78	Panthera_spelaea_U_IMNH.###		
79	Panthera_tigris_F_MNHN.CG1895-355		
80	Panthera_tigris_M_MNHN.CG1985-1860		
81	Panthera_tigris_U_SAP.ANTRO.2954		
82	Panthera_uncia_F_MNHN.CG2016-1664	16, 17	Symmetrization and then estimation using all the other specimens belonging to the genus *Panthera*
83	Panthera_uncia_M_MNHN.CG1998-1248		
84	Pardofelis_marmorata_U_MNHN.CG1886-25		
85	Prionailurus_bengalensis_F_MNHN.CG1954-293		
86	Prionailurus_planiceps_U_MNHN.CG1873-228		
87	Prionailurus_rubiginosus_U_MNHN.CG1872-70	15, 16, 17, 30	Estimated using all the other specimens belonging to the genus *Prionailurus*
88	Prionailurus_viverrinus_F_MNHN.CG2015-1300		
89	Puma_concolor_F_UV.4117		
90	Puma_concolor_M_MNHN.CG1926-250		
91	Puma_concolor_U_IMNH.R-27		
92	Puma_concolor_U_ISM.ZOO.693928		
93	Puma_concolor_U_MZB2003-1534		
94	Smilodon_fatalis_U_F.AM.14349		
95	Smilodon_neogaeus_U_MSMN.V371		
96	Smilodon_populator_U_MNHN-P-957		
97	Therailurus_diastemata_U_NMBS.Rss83		
98	Xenosmilus_hodsonae_U_BC-113		
99	Yoshi_garevskii_U_MMNH-Sk-69	25, 30	Symmetrization and then estimation using all the other specimens belonging to the clade Machairodontinae

Table S5: Missing landmarks and methods of estimation
